# Risk stratification for early-stage NSCLC progression: a federated learning framework with large-small model synergy

**DOI:** 10.3389/fonc.2025.1719433

**Published:** 2025-12-16

**Authors:** Zijun Huang, Bao Feng, Yehang Chen, Liebin Huang, Yuan Chen, Xiaobei Duan, Xiaojuan Chen, Huan Lin, Kunwei Li, Yuping Li, Qiong Li, Xiaohong Ruan, Xiangmeng Chen, Wansheng Long

**Affiliations:** 1 School of Electronic Engineering and Automation, Guilin University of Electronic Technology, Guilin, China; 2 Laboratory of Intelligent Detection and Information Processing, Guilin University of Aerospace Technology, Guilin, China; 3 Jiangmen Key Laboratory of Artificial Intelligence in Medical Image Computation and Application, Jiangmen Central Hospital, Jiangmen, Guangdong, China; 4 Department of Radiology, Jiangmen Central Hospital, Jiangmen, China; 5 Department of Gynecology, Jiangmen Central Hospital, Jiangmen, China; 6 Nuclear Medicine Department, Jiangmen Central Hospital, Jiangmen, China; 7 Department of Radiology, Guangdong General Hospital, Guangzhou, China; 8 Department of Radiology, The Fifth Affiliated Hospital of Sun Yat-sen University, Zhuhai, China; 9 Department of Radiology, Sun Yat-sen University Cancer Center, Guangzhou, China; 10 Clinical Transformation and Application Key Lab for Obstetrics and Gynecology, Pediatrics, and Reproductive Medicine of Jiangmen, Jiangmen Central Hospital, Jiangmen, China

**Keywords:** deep learning, federated communication, large-small model synergy, multiple cross-task validation, NSCLC

## Abstract

**Purpose:**

Accurate prediction of non-small cell lung cancer (NSCLC) progression is crucial for guiding clinical decision-making and alleviating patients’ psychological burden. Overtreatment risks unnecessary trauma for low-risk patients, while undertreatment may delay timely intervention for high-risk cases, worsening prognosis. This study aimed to develop a precise risk stratification system. The aim of this study is to develop an accurate risk stratification system to improve prediction accuracy and stratified management. To validate the proposed framework’s versatility and robustness, we conducted multiple cross-task validation experiments.

**Materials and methods:**

This retrospective study included 926 patients with resected stage I–IIA NSCLC who underwent radical surgery at four centers between January 2014 and September 2019. A multi-center intelligent risk stratification model FesCPI (Federated cross-scale Common–Personal–Interactive learning) was developed. Model performance was evaluated using the area under the receiver operating characteristic curve (AUC), accuracy, sensitivity, specificity, positive predictive value (PPV), and negative predictive value (NPV). Model stability was assessed with five-fold cross-validation, and clinical utility was further validated through decision curve analysis (DCA). Additionally, cross-task experiments for gastric cancer and endometrial cancer were conducted to validate the model’s performance.

**Results:**

FedCPI consistently outperformed clinical stratification ((DeLong test, p < 0.05)) and federated learning baselines across multicenter tasks. In early-stage NSCLC, it achieved AUCs up to 0.9255 and ACCs up to 0.8909, with 4.23–15.95% gains over competing models. The framework continues to demonstrate outstanding performance in various tasks, including predicting gastric cancer recurrence and endometrial cancer infiltration, thereby validating the effectiveness of this methodology. Feature analyses confirmed complementary roles of VFMs and ResNet18, while ablation studies showed that both Large–Small Model Feature Decomposition and Fusion (LMSF) and Federated Adaptive Communication Mechanism (FACM) were indispensable for optimal performance.

**Conclusion:**

Our findings suggest that the DL(deep learning)-based FedCPI framework provides a non-invasive, accurate, and reliable tool for early-stage lung cancer risk stratification. Furthermore, the methodology demonstrated excellent performance in independent validation experiments for clinical tasks involving gastric cancer and endometrial cancer. By improving diagnostic precision, this approach has the potential to optimize clinical decision-making and reduce the burden of overtreatment for patients.

## Introduction

Lung cancer remains the leading cause of cancer-related mortality worldwide, and its burden is particularly severe in China, where the overall 5-year survival rate remains as low as 19.7% (1). Although patients diagnosed at stage I can achieve relatively favorable outcomes with postoperative 5-year survival rates of 77%–92% (2), a substantial proportion still experience disease progression or recurrence despite early intervention. This clinical reality underscores an urgent need for accurate risk stratification tools capable of identifying high-risk individuals at an early stage. Reliable prediction not only reduces diagnostic uncertainty but also helps avoid overtreatment in low-risk patients and undertreatment in high-risk patients, thereby enabling more precise management strategies and improving long-term prognosis.

Computed tomography (CT) is the primary imaging modality for lung cancer screening and diagnosis. While CT demonstrates excellent sensitivity in nodule detection, its diagnostic reliability for risk stratification of progression remains limited. Due to the high overlap in imaging manifestations, radiologists often struggle to accurately distinguish progression risk based solely on CT features (3). Although biopsy or surgery remains the gold standard for definitive diagnosis, these invasive procedures increase clinical burden and risks for patients (4).

In recent years, artificial intelligence (AI), particularly DL, has shown tremendous potential in various lung cancer imaging tasks, including nodule detection, malignancy prediction, and prognostic stratification (5–7). However, most existing studies rely on single-center datasets with limited sample sizes, which increases the risk of overfitting and poor generalizability. To address data scarcity, federated learning (FL) (8, 9) has been proposed to enable collaborative multi-center modeling while preserving data privacy. Nevertheless, data heterogeneity across centers remains a major challenge. Variations in imaging equipment, scanning protocols, annotation strategies, and patient demographics often lead to significant feature distribution shifts (10, 11), undermining the stability and generalizability of global models.

To improve generalization, researchers have attempted to leverage vision foundation models (VFMs) (12), pretrained on large-scale multi-domain datasets, to extract robust and transferable representations (6). However, VFMs often entail high computational cost and complex deployment, limiting their widespread adoption in clinical centers (13). Conversely, lightweight local models offer greater flexibility in deployment and are more sensitive to capturing local diagnostic details (14), but they lack the robust generalization capability of VFMs. Therefore, establishing an effective large–small model synergy—leveraging the cross-domain generalization capacity of VFMs while integrating the fine-grained diagnostic sensitivity and deployability of lightweight models—represents a pivotal step toward precise early-stage lung cancer progression stratification. Furthermore, existing collaborative strategies such as knowledge distillation or simple feature fusion struggle to effectively resolve semantic inconsistencies between large and small models. When integrating diverse representations, simplistic stacking approaches not only dilute discriminative signals but also suppress complementary knowledge (15, 16). Task-independent information may further induce false associations between features (17). More critically, shared common knowledge often contains redundancies that impair models’ ability to capture unique information (18). Against this backdrop, addressing issues at the feature level offers a new breakthrough direction. As the critical bridge between raw image data and predictive models, feature quality directly determines the discriminative capabilities of downstream models.

To address the aforementioned challenges, we propose a large–small model collaborative federated learning paradigm, which simultaneously enables the following: (i) Leveraging the advantages of both large and lightweight models, acquires the generalized semantic representations of large-scale Vision Foundation Models (VFMs) while preserving the discriminative capability and deployability of lightweight local models. (ii) Structured decomposition of features from both large and small models into common, personalized, and interactive subspaces, followed by robust fusion. Through feature disentanglement and dynamic fusion, heterogeneous multi-center data are transformed into effective structured and learnable signals. (iii)An effective multi-center communication mechanism [e.g., Hierarchical Federated Aggregation (19)] is established to selectively coordinate shared knowledge, personalized knowledge, and interactive knowledge across institutions. This federated learning paradigm transforms information discrepancies caused by heterogeneity into structured and learnable signals, enabling multi-center medical modeling to not only maintain cross-institutional robustness but also remain sensitive to fine-grained local diagnostic cues, thereby meeting the dual requirements of global generalization and local adaptation. In addition, independent cross-task validation can demonstrate the validity and universality of the methodology from different tasks.

## Materials and methods

### Description of multicenter datasets

This study retrospectively collected chest CT data from 926 patients with early-stage lung cancer across four independent medical institutions. All participants had solid non-small cell lung cancer (NSCLC) and underwent radical surgical resection between January 2014 and September 2019. All methods were carried out in accordance with relevant guidelines and regulations, including the principles of the Declaration of Helsinki. Given the retrospective nature of the study, the requirement for informed consent was waived by the approving ethics committee. Patients were categorized into two groups: progression(tumor metastasis or recurrence within three years post-surgery) and non-progression (no evidence of metastasis or recurrence within three years). There are five inclusion criteria: (1) Pathologically confirmed NSCLC (solid type, stage I–II) after surgery; (2) Preoperative chest CT within one month before surgery; (3) CT images retrieved from PACS; (4) Regular follow-up for ≥3 years; (5) CT slice thickness ≤1.5 mm. There are seven exclusion criteria: (1) Small cell lung cancer; (2) Neuroendocrine tumors; (3) Metastatic lesions; (4) Non-solid or part-solid nodules; (5) TNM stage III–IV; (6) Missing clinical or follow-up data; (7) Poor-quality CT images. The specific clinical information of the screened data is shown in **Table 1**. Details of data processing are provided in **Supplementary Material S1**.

**Table 1:** Basic patient information.

Center	Set	Disease type	Age (Mean ± Std)	Maximum diameter	Gender		TNM staging system				CEA		Smoking		Lobulation		Spiculation	
					Male	Female	IA	IB	IIA	IIB	Negative	Positive	Absence	Presence	Absence	Presence	Absence	Presence
CenterA (465)	Train (276)	NP-ELC(229)	60.46 ± 10.177	23.6837 ± 13.302	120	109	140	51	6	32	217	12	172	57	185	44	110	119
		P-ELC(47)	62.77 ± 8.911	30.0362 ± 15.9877	34	13	13	16	4	14	34	13	34	13	43	4	21	26
	Test (189)	NP-ELC(154)	59.88 ± 10.408	21.5568 ± 13.036	81	73	103	30	5	16	131	23	115	39-	19	135	93	61
		P-ELC(35)	59.86 ± 10.904	33.2971 ± 13.036	17	18	13	9	3	10	20	15	25	10	7	28	14	21
CenterB (198)	Train (104)	NP-ELC(85)	60.59 ± 9.810	2.7389 ± 1.2802	46	39	39	33	2	8	69	21	61	24	4	81	43	8
		P-ELC(19)	66.11 ± 9.474	2.3037 ± 1.0016	11	8	9	9	2	3	15	4	13	6	2	17	42	11
	Test (94)	NP-ELC(73)	61.37 ± 10.960	2.738 ± 1.4667	45	28	31	25	1	11	60	13	51	22	11	62	38	35
		P-ELC(21)	64.52 ± 11.259	3.678 ± 2.074	15	6	9	9	2	6	10	11	14	7	3	18	4	17
CenterC (148)	Train (78)	NP-ELC(62)	58.21 ± 10.238	58.21 ± 10.238	38	24	33	14	4	7	50	12	36	26	11	51	41	10
		P-ELC(16)	56.44 ± 12.806	56.44 ± 12.806	9	7	9	3	2	6	13	3	10	6	2	14	21	6
	Test (70)	NP-ELC(59)	58.153 ± 10.171	24.149 ± 13.474	26	33	41	7	0	8	47	12	35	24	10	49	37	22
		P-ELC(11)	58.00 ± 9.241	27.255 ± 12.089	7	4	3	5	3	3	8	3	5	6	2	9	7	4
CenterD (115)	Train (60)	NP-ELC(50)	58.86 ± 12.3189	19.48 ± 6.3221	26	24	35	13	1	1	41	9	33	17	0	50	0	50
		P-ELC(10)	61.80 ± 6.6131	19.4 ± 3.8644	6	4	5	4	0	1	7	3	5	5	0	10	0	10
	Test (55)	NP-ELC(47)	59.36 ± 9.586	19.936 ± 6.654	24	23	33	13	0	1	36	11	34	13	0	47	0	47
		P-ELC(8)	57.75 ± 4.950	19.875 ± 6.7281	4	4	6	**1**	**1**		6	2	6	2	0	8	0	8

NP-ELC, non-progression early-stage lung cancer; P-ELC, progression early-stage lung cancer; Mean value, Std standard deviation.

### Overview of the FedCPI framework

**Figure 1:**
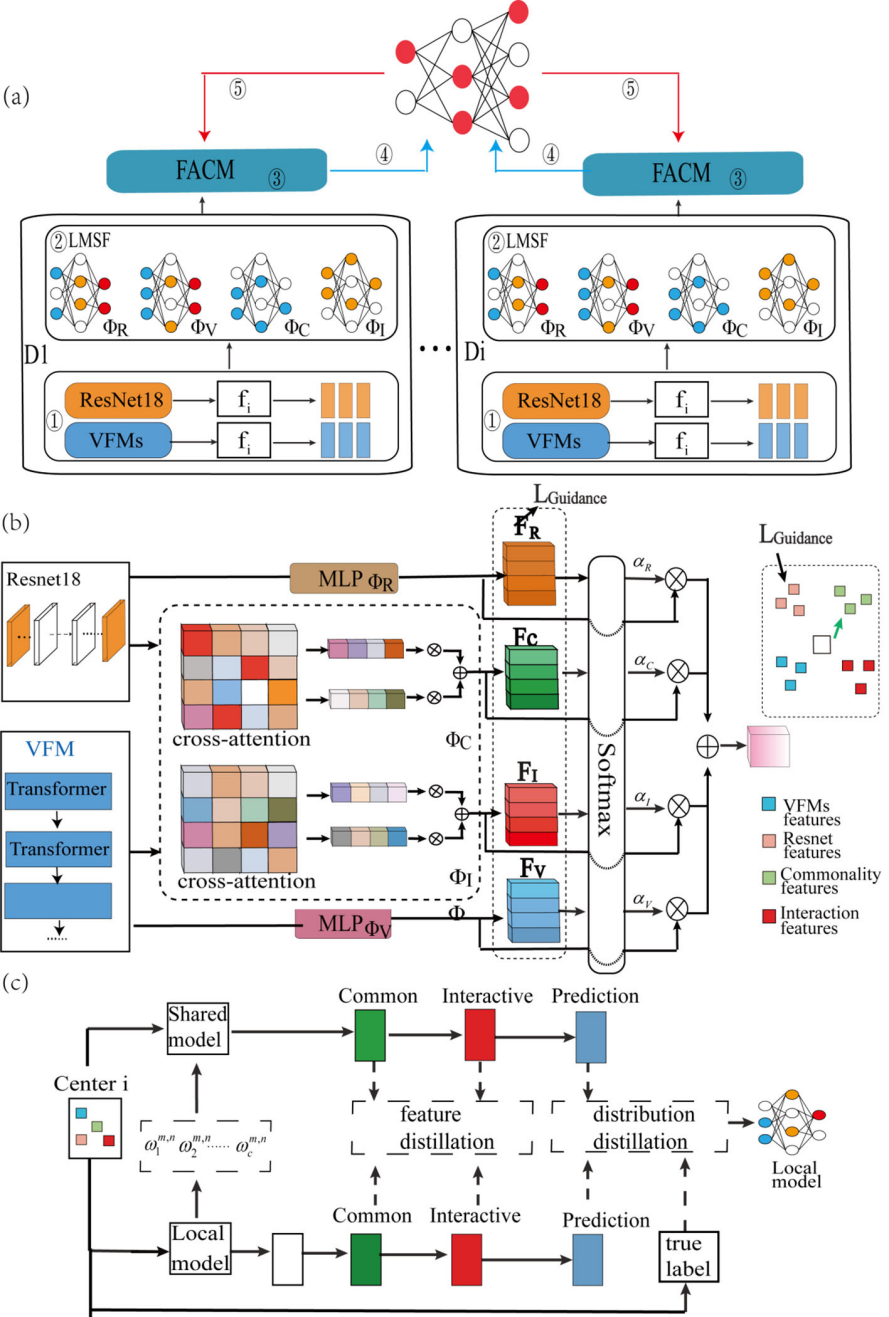
Overview of the FedCPI framework and module details. **(a)** Federated learning architecture: Illustrates the collaborative workflow between a central server and multiple distributed hospital clients. Clients train models on local data and only upload model updates (not raw data) to the server for secure aggregation. The global model is then broadcast back to clients, forming a closed loop. **(b)** Large-Small Model feature decomposition and Fusion (LMSF) module: Details the on-client processing mechanism. Features (f_i) from a large Vision Foundation Model (VFM) and a lightweight local model (ResNet18) are interacted and aligned via a Cross-Attention mechanism, and decomposed into common, personalized, and interactive feature subspaces. They are then projected and fused by Multi-Layer Perceptrons (MLPs, Φ) to form a more discriminative unified representation. **(c)** Federated Adaptive Communication Mechanism (FACM): Depicts the differentiated aggregation strategies on the server for different feature subspaces. It employs global averaging for common features, local retention for personalized features, and performance-driven weighting for interactive features, achieving an optimal balance between global consistency and local adaptability.

The paper propose FesCPI (Federated cross-scale Common–Personal–Interactive learning), a federated learning framework, as shown in **Figure 1**. FesCPI is composed of two main modules: Large–Small Model Feature Decomposition and Fusion (LMSF) and Federated Adaptive Communication Mechanism (FACM). Together, these modules transform heterogeneity-induced discrepancies into structured and learnable signals, enhancing robustness and interpretability in early lung cancer risk stratification.

### Large–small model feature decomposition and fusion

The LMSF module enables consistent and complementary feature representations by aligning, decomposing, and fusing multi-scale knowledge from large-scale Vision Foundation Models (VFMs) and lightweight local backbones [ResNet18 (20)]. The Vision Foundation Model (VFM) we used is dinov2_vitb14. To mitigate semantic inconsistency, we introduce a lightweight alignment loss to shrink the representational gap between VFMs and local models.

The aligned features are further decomposed into three structured subspaces: (1) Common (C), capturing stable and consistent features shared by VFMs and ResNet18; (2) Personal (P), reflecting model-specific expressive capacities; and (3) Interactive (I), encoding complementary relations and semantic couplings across models. To further enhance the discriminative ability of these subspaces, LMSF introduces a queue-based contrastive learning strategy, improving intra-class consistency and inter-class separability. In addition, a lightweight gating network performs dynamic weighting to balance the contributions of each subspace.

### Federated adaptive communication mechanism

FACM performs subspace-aware aggregation across centers. Common subspace (C): aggregated via global averaging, promoting generalization. Personal subspace (P): retained locally, ensuring sensitivity to center-specific patterns. Interactive subspace (I): aggregated with performance-driven dynamic weighting, balancing local and global knowledge. After aggregation, the global encoder serves as a teacher model to provide module-level knowledge distillation for local models, strengthening globally generalizable features while alleviating overfitting to local peculiarities. In addition, the lightweight ResNet18 backbone is also aggregated and redistributed to local clients. This design ensures that the globally integrated knowledge can be directly mapped to a deployable local backbone, rather than only existing in the decomposed subspaces.

By integrating LMSF and FACM, FesCPI transforms heterogeneity and semantic inconsistency into structured and learnable signals. This enables federated modeling that (i) leverages the generalization capacity of VFMs, (ii) preserves the lightweight adaptability of local models, and (iii) achieves robust and interpretable cross-center early lung cancer risk stratification. Detailed model configurations and procedures are provided in the **Supplementary Materials S2, S3**.

## Result summary

### Early lung cancer progression screening

As the first case study, we collected early-stage lung cancer patient data from four independent medical institutions, comprising a total of 926 cases. For each center, the samples were split into training and test sets at a 5:5 ratio using adversarial validation (21). Based on the FedCPI framework, we conducted local model training at each center and achieved promising test performance. The area under the ROC curve (AUC)values (22) on the test sets of the four centers were 0.8276, 0.8743, 0.8883, and 0.9255, respectively.

**Figure 2:**
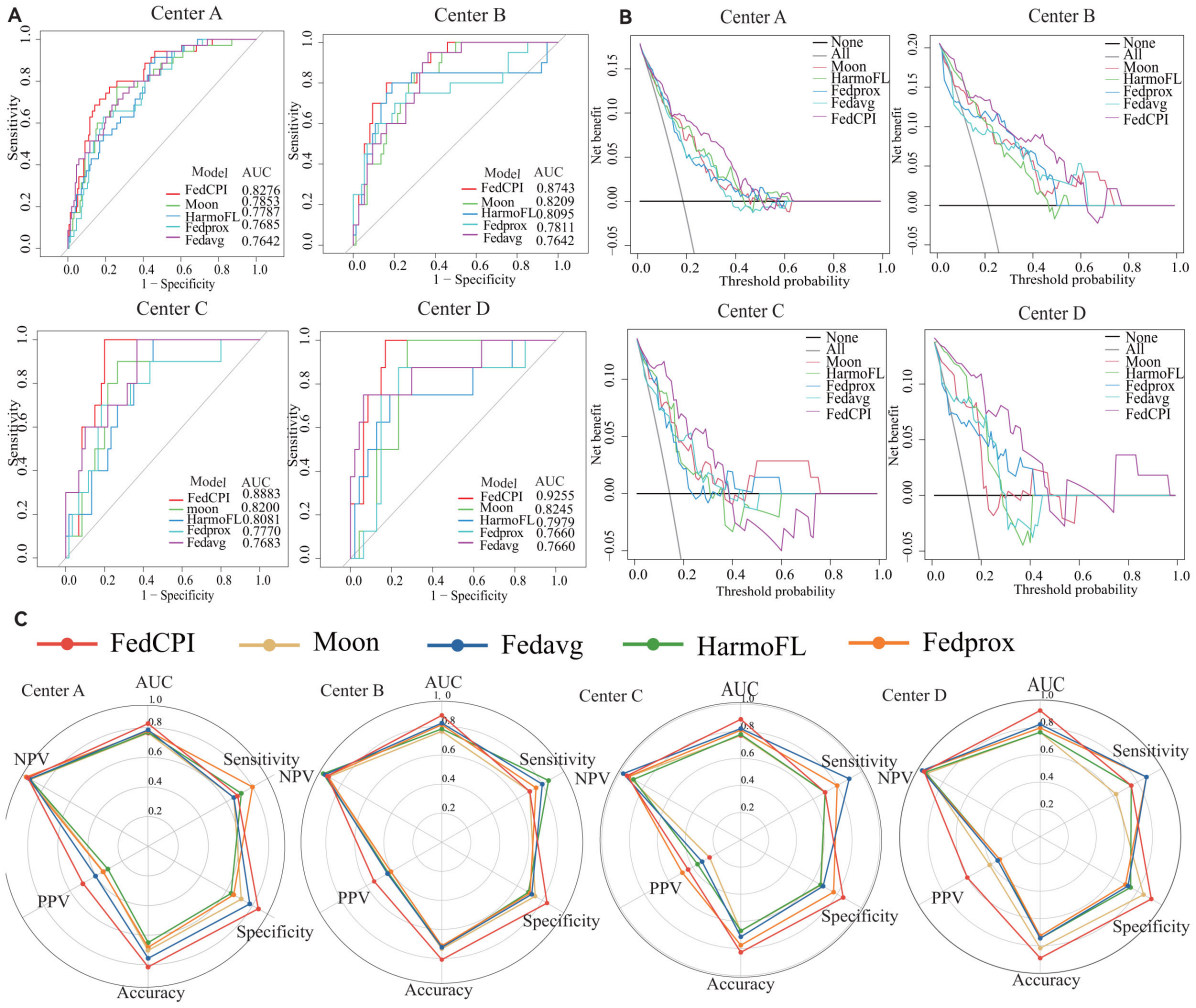
ROC curves, DCA curves and radar charts for the four centers. **(A)** ROC curves of five models in four centers. **(B)** DCA curves of five models in four centers. **(C)** Radar chart comparison of five models in four centers. Federated cross-scale Common–Personal–Interactive learning, Early Lung Cancer, AUC Area Under the Curve,TPR True Positive Rate, FPR False Positive Rate, PPV Positive Predictive Value, NPV C Radar chart comparison of five models in four centers.

To evaluate the diagnostic performance of FedCPI, we compared it against four federated learning baselines, namely FedAvg (23), FedProx (24), HarmoFL (25), and MOON (26). The results demonstrated that FedCPI achieved average AUC improvement rates of 5.34%, 8.04%, 9.50%, and 13.69% across the different centers. In addition, FedCPI attained an overall prediction accuracy of 0.826 (337/408) for distinguishing progression from non-progression in early-stage lung cancer, thereby improving early diagnosis and enabling timely personalized prognosis. **Figure 2** presents the ROC and decision curve analysis (DCA) curves (27) of the five methods across the four centers. As shown in radar charts (28), FedCPI consistently achieved the highest overall performance and net clinical benefit across all four centers. More detailed results can be found in **Supplementary S4**.

**Figure 3:**
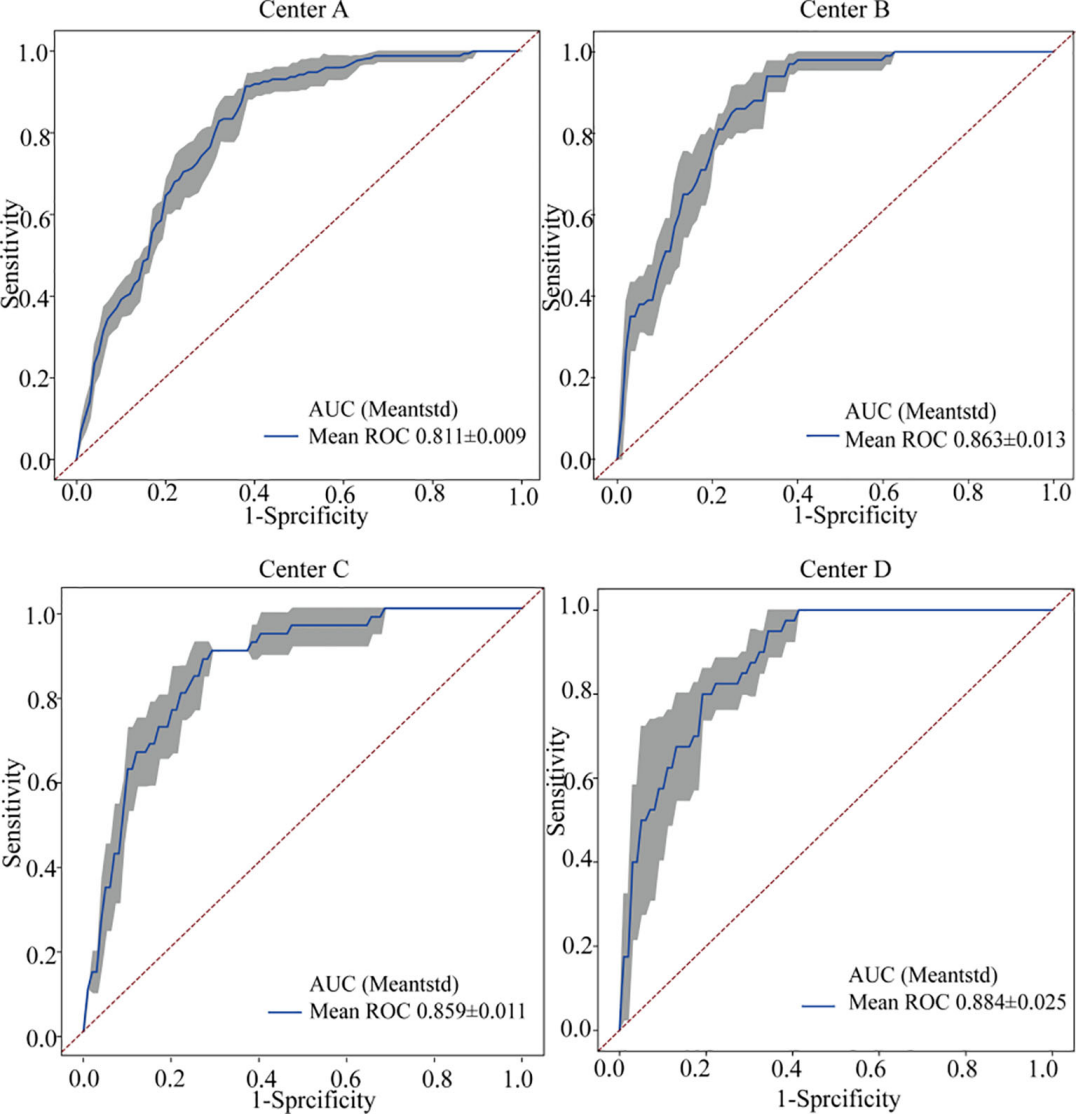
The fivefold cross-validation ROC curves of the four centers. The blue curve represents the average AUC of the fivefold curve. The gray area indicates the upper and lower limits of the ROC curve, with the error bands within this gray area representing the boundaries of the fivefold cross-validation ROC curves. The average AUC and standard deviation for threefold cross-validation are also shown.

To further assess the robustness of FedCPI, conducted five-fold cross-validation (29) using the early-stage lung cancer progression case as a reference. As shown in **Figure 3**, the average cross-validation AUC across the four centers were 0.811 ± 0.009, 0.863 ± 0.013, 0.859 ± 0.011, and 0.884 ± 0.025, confirming the model’s stability.

**Figure 4:**
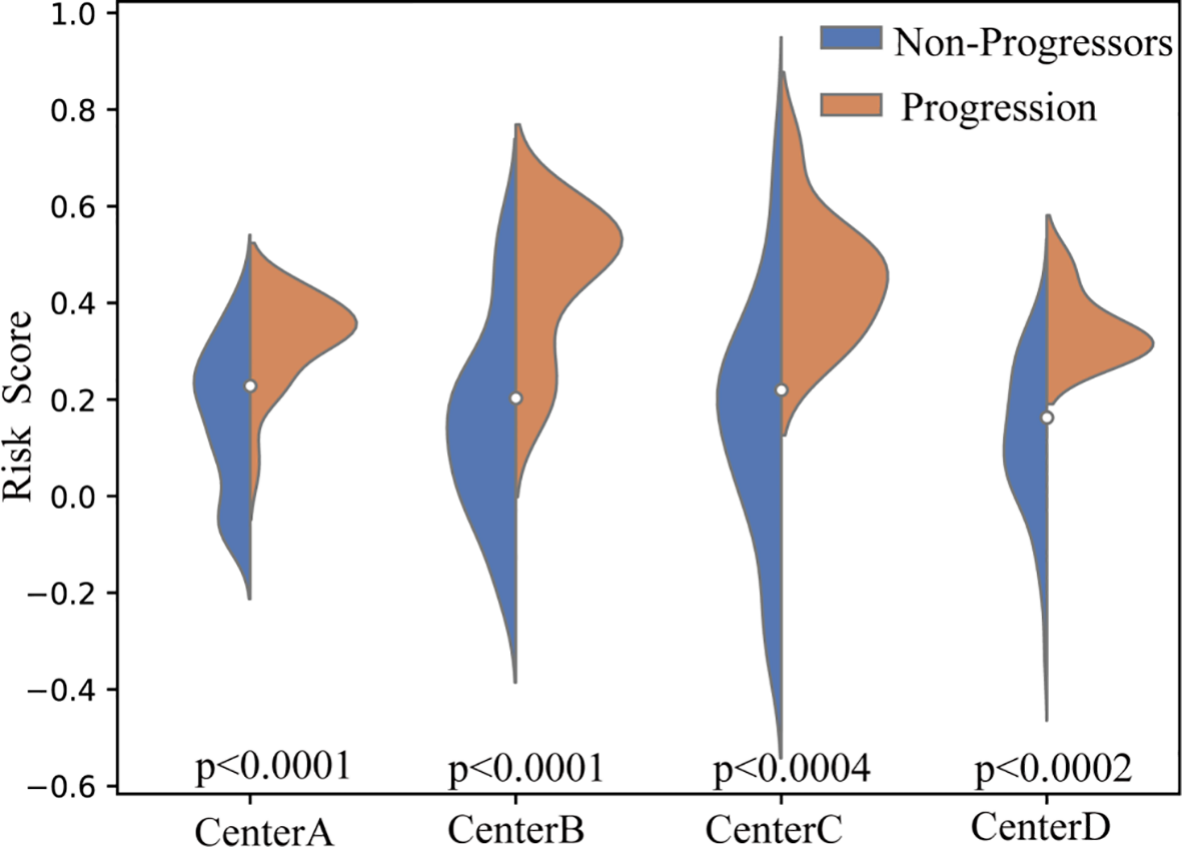
Violin plots of predicted risk score scores across four independent test cohorts. Blue indicates NP-risk score patients, and orange indicates P-risk score patients. Across all centers (A–D), P-risk score patients consistently demonstrated significantly higher risk score scores compared to NP-risk score patients (p < 0.0001 for Centers A and B, p < 0.0004 for Center C, and p < 0.0002 for Center D).

To further evaluate the discriminative ability of the proposed framework, we compared the distribution of the predicted risk score scores between the P-risk score and NP-risk score groups across all independent test cohorts. As illustrated in the violin plots (**Figure 4**), the risk score scores of P-risk score patients were consistently and significantly higher than those of NP-risk score patients across four centers (Center A, B, C, and D). Statistical testing confirmed robust between-group separation in all cohorts (p < 0.0001 for Center A and B, p < 0.0004 for Center C, and p < 0.0002 for Center D). The distinct median values and non-overlapping interquartile ranges further demonstrate the stability of this separation. These findings highlight that the proposed model achieves reliable and consistent patient stratification across heterogeneous centers.

### Comparison with traditional clinical models

**Table 2** summarizes the comparative performance of the proposed FedCPI framework against existing clinical predictors across four independent centers. These predictors include: univariable logistic regression models based on TNM staging, histological features (lobulation and spiculation), and maximum lesion diameter, as well as a multivariable logistic regression model incorporating other clinical factors. In the primary task of early-stage non-small cell lung cancer risk stratification, FedCPI demonstrated excellent and stable predictive performance across all four independent medical centers. FedCPI significantly outperformed traditional clinical models in terms of AUC values (Center A: 0.8276, Center B: 0.8743, Center C: 0.8883, Center D: 0.9255) and accuracy rates (Center A: 0.8148, Center B: 0.8085, Center C: 0.8143, Center D: 0.8909) across all centers. Notably, while maintaining high specificity (ranging from 0.8378 to 1.000 across centers), FedCPI achieved substantially improved sensitivity (ranging from 0.2500 to 0.7000 across centers)—a balance that is critically important in clinical practice. It successfully overcomes the limitations of single-center models and traditional clinical indicators, offering a significant improvement in predictive accuracy for early-stage non-small cell lung cancer patients in a federated learning setting.

**Table 2:** Comparison with traditional clinical models.

Center	Algorithm	AUC	Accuracy	Sensitivity	Specificity	PPV	NPV
A (JM)	TNM Staging System	0.6675 [0.571-0.764]	0.7725 [0.708-0.826] (146/189)	0.3714 [0.232-0.537] (13/35)	0.8636 [0.801-0.909] (133/154)	0.3824 [0.239-0.550] (13/34)	0.8581 [0.794-0.904] (133/155)
	Lobulation	0.4617 [0.390-0.534]	0.8148 [0.753-0.864] (154/189)	0.0000 [0.000-0.099] (0/35)	1.0000 [0.976-1.000] (154/154)		0.8148 [0.753-0.864] (154/189)
	Spiculation	0.6019 [0.511-0.693]	0.8148 [0.753-0.864] (154/189)	0.0000 [0.000-0.099] (0/35)	1.0000 [0.976-1.000] (154/154)		0.8148 [0.753-0.864] (154/189)
	Maximum diameter	0.7186 [0.619-0.818]	0.6984 [0.630-0.759] (132/189)	0.7143 [0.549-0.837] (25/35)	0.6948 [0.618-0.762] (107/154)	0.3472 [0.248-0.462] (25/72)	0.9145 [0.850-0.953] (107/117)
	MLR	0.7549 [0.672-0.838]	0.7143 [0.646-0.774] (135/189)	0.6000 [0.436-0.744] (21/35)	0.7403 [0.666-0.803] (114/154)	0.3443 [0.237-0.470] (21/61)	0.8906 [0.825-0.934] (114/128)
	FedCPI	0.8276 [0.755-0.900]	0.8148 [0.753-0.864] (154/189)	0.6857 [0.520-0.814] (24/35)	.8442 [0.779-0.893] (130/154)	0.5000 [0.364-0.636] (24/48)	0.9220 [0.866-0.956] (130/141)
B (SY)	TNM Staging System	0.5764 [0.452-0.701]	0.6702 [0.570-0.757] (63/94)	0.2308 [0.110-0.421] (6/26)	0.8382 [0.733-0.907] (57/68)	0.3529 [0.173-0.587] (6/17)	0.7403 [0.633-0.825] (57/77)
	Lobulation	0.5305 [0.453-0.608]	0.7234 [0.626-0.804] (68/94)	0.0000 [0.000-0.129] (0/26)	1.0000 [0.947-1.000] (68/68)		0.7234 [0.626-0.804] (68/94)
	Spiculation	0.6493 [0.547-0.751]	0.7234 [0.626-0.804] (68/94)	0.0000 [0.000-0.129] (0/26)	1.0000 [0.947-1.000] (68/68)		0.7234 [0.626-0.804] (68/94)
	Maximum diameter	0.6456 [0.522-0.769]	0.3830 [0.291-0.484] (36/94)	0.8846 [0.710-0.960] (23/26)	0.1912 [0.115-0.300] (13/68)	0.2949 [0.205-0.404] (23/78)	0.8125 [0.570-0.934] (13/16)
	MLR	0.6199 [0.498-0.742]	0.6170 [0.516-0.709] (58/94)	0.6538 [0.462-0.806] (17/26)	0.6029 [0.484-0.711] (41/68)	0.3864 [0.257-0.534] (17/44)	0.8200 [0.692-0.902] (41/50)
	FedCPI	0.8743 [0.798-0.951]	0.8085 [0.717-0.875] (76/94)	0.7000 [0.481-0.855] (14/20)	0.8378 [0.738-0.905] (62/74)	0.5385 [0.355-0.712] (14/26)	0.9118 [0.821-0.959] (62/68)
C (ZD)	TNM Staging System	0.7449 [0.612-0.878]	0.7286 [0.615-0.819] (51/70)	0.2143 [0.076-0.476] (3/14)	0.8571 [0.743-0.926] (48/56)	0.2727 [0.097-0.566] (3/11)	0.8136 [0.696-0.893] (48/59)
	Lobulation	0.5357 [0.409-0.662]	0.8000 [0.692-0.877] (56/70)	0.0000 [0.000-0.215] (0/14)	1.0000 [0.936-1.000] (56/56)		0.8000 [0.692-0.877] (56/70)
	Spiculation	0.4643 [0.326-0.602]	0.8000 [0.692-0.877] (56/70)	0.0000 [0.000-0.215] (0/14)	1.0000 [0.936-1.000] (56/56)		0.8000 [0.692-0.877] (56/70)
	Maximum diameter	0.6824 [0.490-0.874]	0.4571 [0.346-0.573] (32/70)	0.7857 [0.524-0.924] (11/14)	0.3750 [0.260-0.506] (21/56)	0.2391 [0.139-0.379] (11/46)	0.8750 [0.690-0.957] (21/24)
	MLR	0.6696 [0.484-0.855]	0.7571 [0.645-0.842] (53/70)	0.5714 [0.326-0.786] (8/14)	0.8036 [0.682-0.887] (45/56)	0.4211 [0.231-0.637] (8/19)	0.8824 [0.766-0.945] (45/51)
	FedCPI	0.8883 [0.812-0.965]	0.8143 [0.708-0.888] (57/70)	0.6000 [0.313-0.832] (6/10)	0.8500 [0.739-0.919] (51/60)	0.4000 [0.198-0.643] (6/15)	0.9273 [0.827-0.971] (51/55)
D (ZS)	TNM Staging System	0.4907 [0.300-0.681]	0.8545 [0.738-0.924] (47/55)	0.1250 [0.022-0.471] (1/8)	0.9787 [0.889-0.996] (46/47)	0.5000 [0.095-0.905] (1/2)	0.8679 [0.752-0.935] (46/53)
	Lobulation	0.5000	0.8545 [0.738-0.924] (47/55)	0.0000 [0.000-0.324] (0/8)	1.0000 [0.924-1.000] (47/47)		0.8545 [0.738-0.924] (47/55)
	Spiculation	0.5000	0.8545 [0.738-0.924] (47/55)	0.0000 [0.000-0.324] (0/8)	1.0000 [0.924-1.000] (47/47)		0.8545 [0.738-0.924] (47/55)
	Maximum diameter	0.4920 [0.266-0.718]	0.4000 [0.281-0.532] (22/55)	0.5000 [0.215-0.785] (4/8)	0.3830 [0.258-0.526] (18/47)	0.1212 [0.048-0.273] (4/33)	0.8182 [0.615-0.927] (18/22)
	MLR	0.4215 [0.166-0.677]	0.4364 [0.314-0.567] (24/55)	0.3750 [0.137-0.694] (3/8)	0.4468 [0.314-0.588] (21/47)	0.1034 [0.036-0.264] (3/29)	0.8077 [0.621-0.915] (21/26)
	FedCPI	0.9255 [0.855-0.996]	0.8909 [0.782-0.949] (49/55)	0.2500 [0.071-0.591] (2/8)	1.0000 [0.924-1.000] (47/47)	1.0000 [0.342-1.000] (2/2)	0.8868 [0.774-0.947] (47/53)

The confidence interval is omitted due to the model’s poor discriminative ability compared to conventional clinical imaging features (e.g., lobulation and spiculation) in certain centers (AUC ≈ 0.5, confidence interval unestimable). The absence of PPV value occurs when the model fails to predict any positive cases.

To fully evaluate the clinical utility of the FedCPI framework, we conducted a rigorous statistical comparison against key baseline models (**Table 3**). The assessment was performed using DeLong’s test for discrimination improvement, Net Reclassification Improvement (NRI), and Integrated Discrimination Improvement (IDI), with p-values adjusted for False Discovery Rate (P_FDR). The results unequivocally demonstrate that FedCPI achieves statistically significant and clinically relevant superior performance over traditional clinical indicators. FedCPI exhibited significantly enhanced discrimination ability compared to most benchmarks, as evidenced by DeLong’s test. This was consistently observed against the TNM Staging System in Centers A, B, and D (p < 0.01), and against both Lobulation and Spiculation across all centers (p < 0.001). While the improvement over MLR and Maximum Diameter was more variable across centers, the trend favored FedCPI, reaching significance in multiple cohorts. The NRI analysis revealed that FedCPI provides a substantial and consistent improvement in patient risk stratification. The improvements were highly significant (p < 0.001) across nearly all head-to-head comparisons in Centers B and D. The IDI results confirmed that FedCPI offers a robust and holistic upgrade in predictive performance. The improvements were universally highly significant (p < 0.001) against all comparator models in Centers B, C, and D, and largely significant in Center A. This indicates that FedCPI not only improves ranking (NRI) but also achieves a net increase in separation between the predicted probability distributions for events and non-events. Additional algorithm comparison results are presented in **Supplementary Table S4** of **Supplementary Materials**.

**Table 3:** Model difference analysis.

FedCIP clinical models			MLR	TNM staging system	Lobulation	Spiculation	Maximum diameter
FedCIP	Center A	DeLong P_FDR	1.9313 (p=0.080)	2.877 (p=0.009)	6.475 (p<0.001)	3.653 (p<0.001)	1.326 (p=0.2309)
		NRI P_FDR	2.359 (p=0.022)	2.739 (p=0.1333)	7.189 (p<0.001)	7.081 (p<0.001)	3.667 (p<0.001)
		IDI P_FDR	2.211 p=0.0340	4.287 p<0.001	7.742 p<0.001	8.056 (p<0.001)	5.070 (p<0.001)
	Center B	DeLong P_FDR	3.356 p=0.0027	3.884 p<0.001	5.929 p<0.001	3.562 p=0.0018	3.317 p=0.0027
		NRI P_FDR	3.390 p<0.001	6.548 p<0.001	5.298 p<0.001	6.081 p<0.001	5.406 p<0.001
		IDI P_FDR	4.906 p<0.001	6.759 p<0.001	7.252 p<0.001	7.441 p<0.001	6.245 p<0.001
	Center C	DeLong P_FDR	1.809 p=0.1320	1.256 p=0.2616	3.827 p<0.001	4.538 p<0.001	1.603 p=0.1632
		NRI P_FDR	2.240 p=0.057	2.531 p=0.0525	11.297 p<0.001	11.622 p<0.001	4.591 p=0.01
		IDI P_FDR	3.792 p<0.001	3.912 p<0.001	5.359 p<0.001	5.660 p<0.001	4.577 p<0.001
	Center D	DeLong _P_FDR	2.554 P=0.0319	4.576 p<0.001	11.858 p<0.001	11.858 p<0.001	3.819 p<0.001
		NRI _P_FDR	8.241 p<0.001	7.518 p<0.001	8.243 p<0.001	8.506 p<0.001	8.185 p<0.001
		IDI _P_FDR	5.395 p<0.001	6.193 p<0.001	6.769 p<0.001	6.930 p<0.001	6.888 p<0.001

In conclusion, this multi-faceted statistical evaluation confirms that the FedCPI framework delivers a definitive advantage over conventional clinical models. It provides a more accurate, reliable, and clinically actionable tool for risk stratification.

### Correlation analysis of features before feature fusion

To validate this hypothesis, performed correlation heatmap (30) analysis on the features output by both models. In the FedCPI framework, large models (VFMs) and small models (ResNet-18) undertake distinct information extraction roles, a division of labor quantitatively validated by our cross-center correlation analysis. As hypothesized, large models, owing to their higher parameter capacity, excel at capturing consistent cross-sample and cross-domain patterns, thereby extracting stable global common features. This is evidenced by a significantly positive average cross-center correlation of 0.32 (± 0.23) for common features, with a high proportion of significant feature pairs (60%). Conversely, small models, with their lightweight structures and stronger adaptability, are better at capturing task- or individual-specific variations, forming localized personalized features. This is confirmed by their significantly lower and slightly negative average cross-center correlation (-0.21 ± 0.16), with only 28% of feature pairs being significant. A formal statistical comparison (t-statistic = 9.20, p < 0.001) conclusively demonstrates that common features is significantly higher than that of personalized features. This stark contrast provides definitive quantitative proof that the two feature types originate from fundamentally different data distributions and serve distinct purposes.

**Figure 5:**
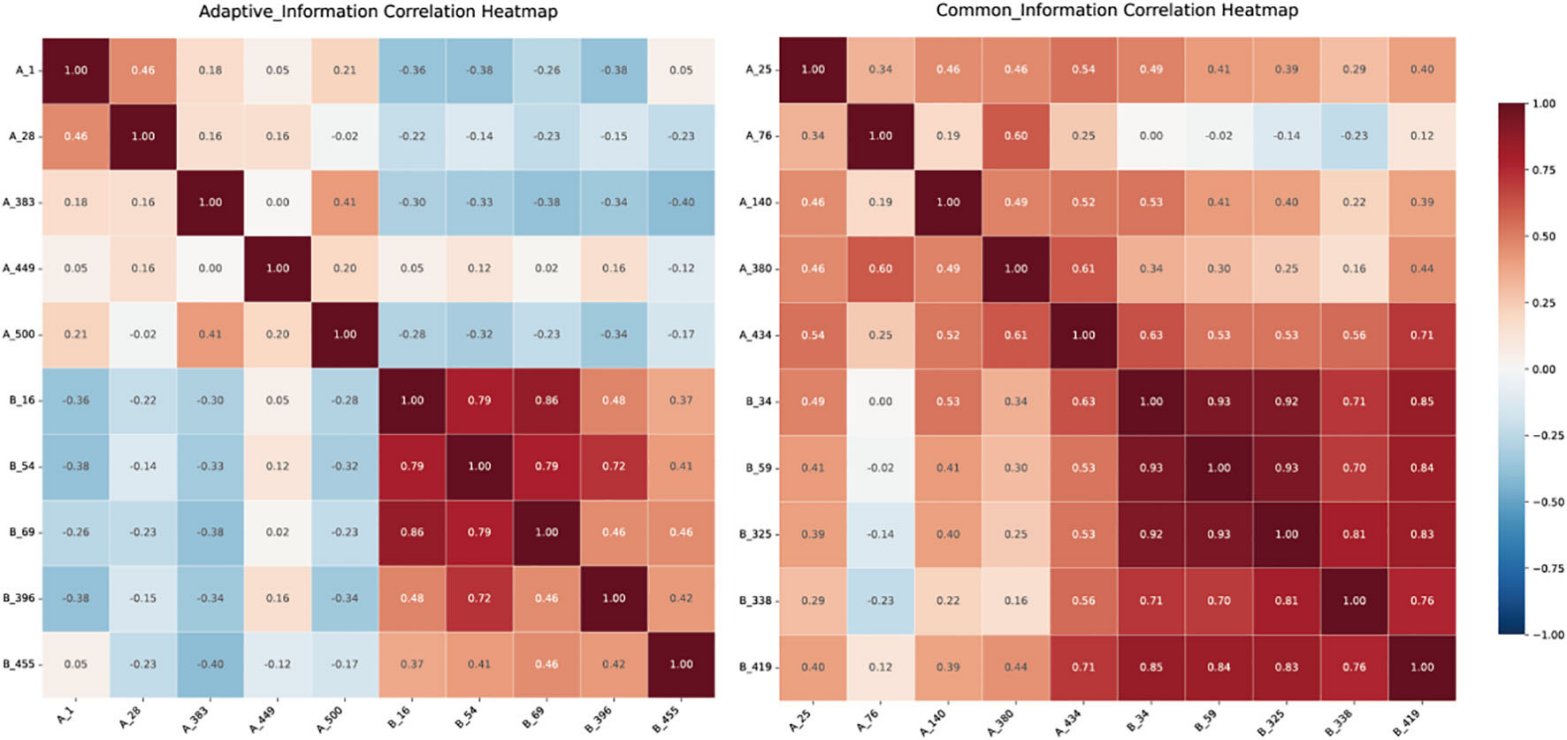
Group A and Group B correspond to the features of the small model (ResNet) and the large model (VFMs).

This statistical evidence aligns perfectly with the visual patterns observed in the correlation heatmaps of **Figure 5**. In the heatmaps corresponding to Common Information, intra-group correlations are generally high, indicating strong stability and internal consistency—a visual reflection of the high cross-center correlation. In contrast, the features in the Personalized Information heatmaps show lower internal consistency, mirroring their low cross-center correlation. Furthermore, the generally low inter-group correlations between the two sets, including some negative values, visually underscore their substantial differences and complementarity.

In conclusion, the combined evidence from rigorous statistical testing and visualization robustly confirms the designed division of labor: large models extract highly stable, globally consistent common features, while small models capture variable, center-specific localized features. This clear separation ensures that the FedCPI model exhibits both remarkable stability and transparent structural interpretability.

### Feature subspace distribution after CPI decomposition

**Figure 6:**
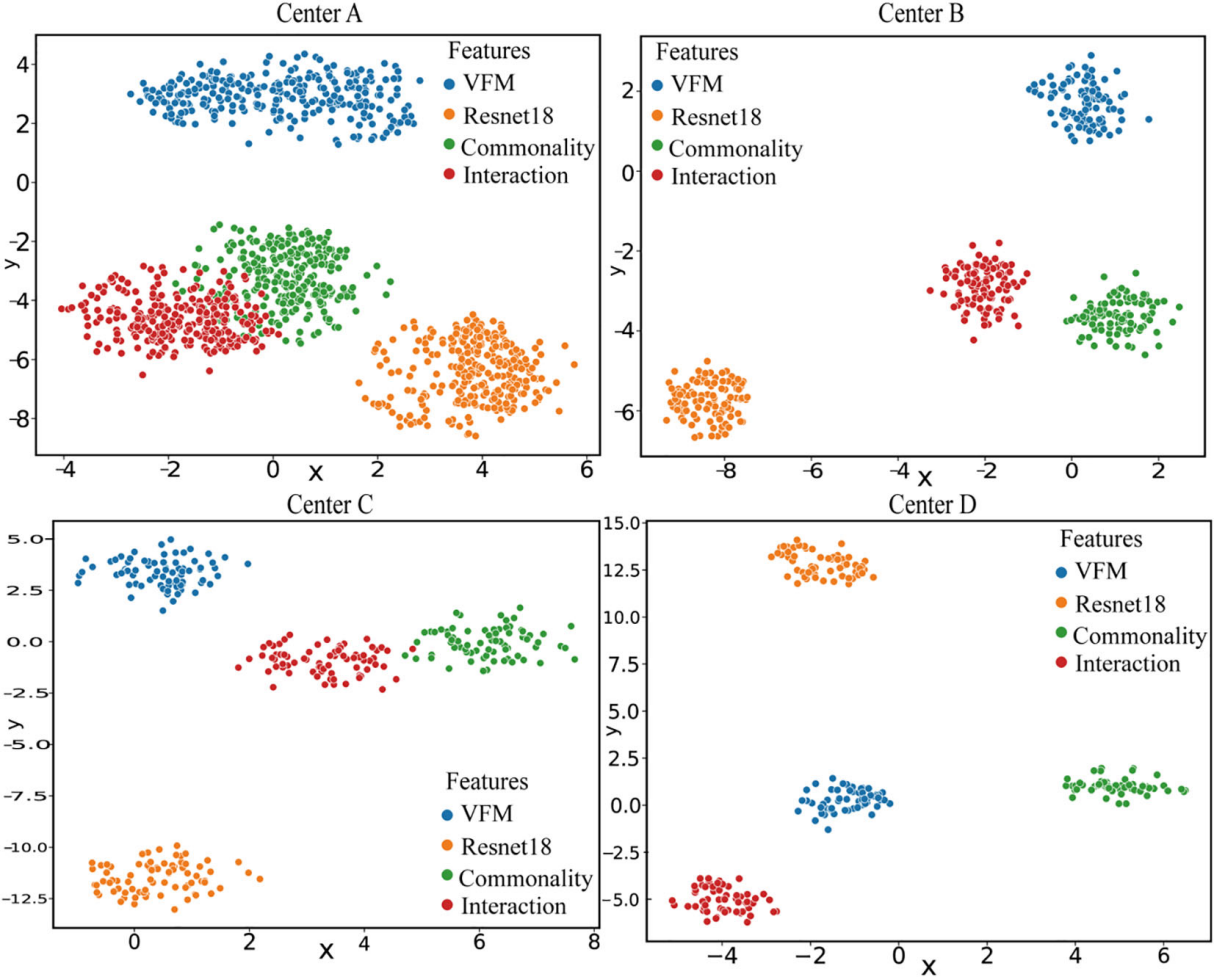
Presents the t-SNE distributions of four subspace features across four centers after CPI-based feature decomposition.

**Figure 6** presents the t-SNE distribution of the four subspace features derived from CPI-based feature decomposition across the four centers. The visualization qualitatively reveals distinct clustering patterns. To quantitatively validate these observations, we computed internal clustering metrics—Silhouette Coefficient, Calinski-Harabasz Index, and Davies-Bouldin Index—for the feature set from Center A (n=276), which exhibited the most pronounced clustering effect. A Silhouette Coefficient of 0.926 indicates highly dense and well-separated clusters; an extremely high Calinski-Harabasz Index (54,028.25) signifies very strong separation between clusters relative to within-cluster variance; and a very low Davies-Bouldin Index (0.104) further confirms the compactness and distinctiveness of the clusters. These metrics collectively provide quantitative evidence for “excellent, highly structured clustering,” confirming that these four feature types form clusters with clear boundaries, distinct separation, and minimal redundancy. Datasets with larger sample sizes show significantly separable feature clusters (high Silhouette score, low DBI), whereas centers with smaller samples exhibit weaker feature clustering. However, by comparing the x and y axes across subplots, the blue (VFMs) and orange (ResNet-18) features show significant variation across centers: distributions for centers A and B are close in some cases, while showing large disparities in others, indicating that personalized features are highly center-dependent. This phenomenon supports the strategy of retaining or fine-tuning these features locally. The red interaction features tend to form clusters across centers while maintaining local independence, suggesting that complementary collaborative features maintain consistency while preserving center-specific differences. This indicates that interaction features are not suitable for direct averaging and require alternative aggregation strategies.

### Correlation after knowledge decomposition and before fusion

**Figure 7:**
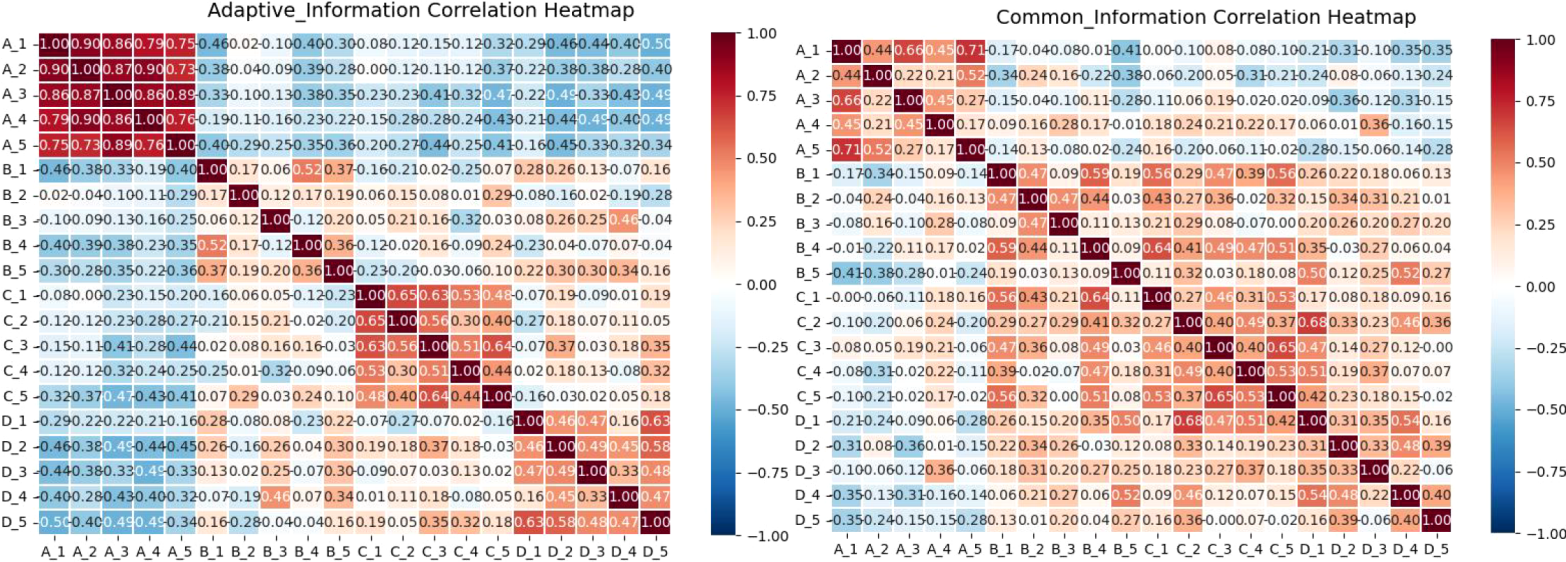
Illustrates the correlation between four central feature categories in early-stage lung cancer. Group A represents features from the large model VFMs; Group B shows features from the small model ResNet18; Group C demonstrates interaction features between the models; and Group D highlights shared features between the models.

As shown in **Figure 7**, the correlation patterns provide clear visual evidence for the distinct roles of different feature types. To quantitatively validate these observations, we conducted rigorous statistical comparisons between the correlation matrices of common and personalized features. The results revealed a significant statistical difference between them, with common features showing a mean correlation of -0.016 ± 0.502 while personalized features exhibited a mean correlation of -0.003 ± 0.451. This difference was statistically significant in the paired t-test (t = -3.69, p = 2.40e-04) though not in the Wilcoxon test (p = 0.380), with a small effect size (Cohen’s d = -0.027). The statistical significance despite the small effect size likely reflects the substantial statistical power from the large sample size (n = 1,081 valid data points).

Further analysis of correlation strength distribution reveals crucial insights: while both feature types show nearly equal proportions of positive correlations (approximately 50%), they differ markedly in their strong correlation patterns. Personalized features exhibit substantially higher proportions of both strong positive (>0.5: 23.86%) and strong negative (<-0.5: 23.60%) correlations, indicating their diverse and polarized nature. In contrast, common features show minimal strong correlations in either direction (approximately 2.5% each for strong positive and negative correlations), confirming their more uniform and stable characteristics. This statistical evidence solidifies the visual interpretations: Within Group A (VFMs features), the low proportion of strong correlations and stable distribution confirm their consistency. Within Group B (ResNet features), the high proportion of strong positive and negative correlations reflects greater variability and polarization, which are defining properties of personalized features.

Critically, the distinct correlation patterns between the two feature types strongly suggest that the two models capture complementary rather than redundant information. Such diversity in correlation patterns is a positive signal in cross-model collaboration, indicating essential feature variation. Interactive features (C) serve as a bridge between common and personalized spaces. They inherit stability from the common features (D) while maintaining adaptability with personalized features (A and B). This shows that the fusion process successfully balances stability with diversity. Common features (D), by contrast, exhibit consistent correlation patterns and minimal strong correlations with A and B, reflecting their role as shared universal representations.

### Cross-center feature subspace relationships

**Figure 8:**
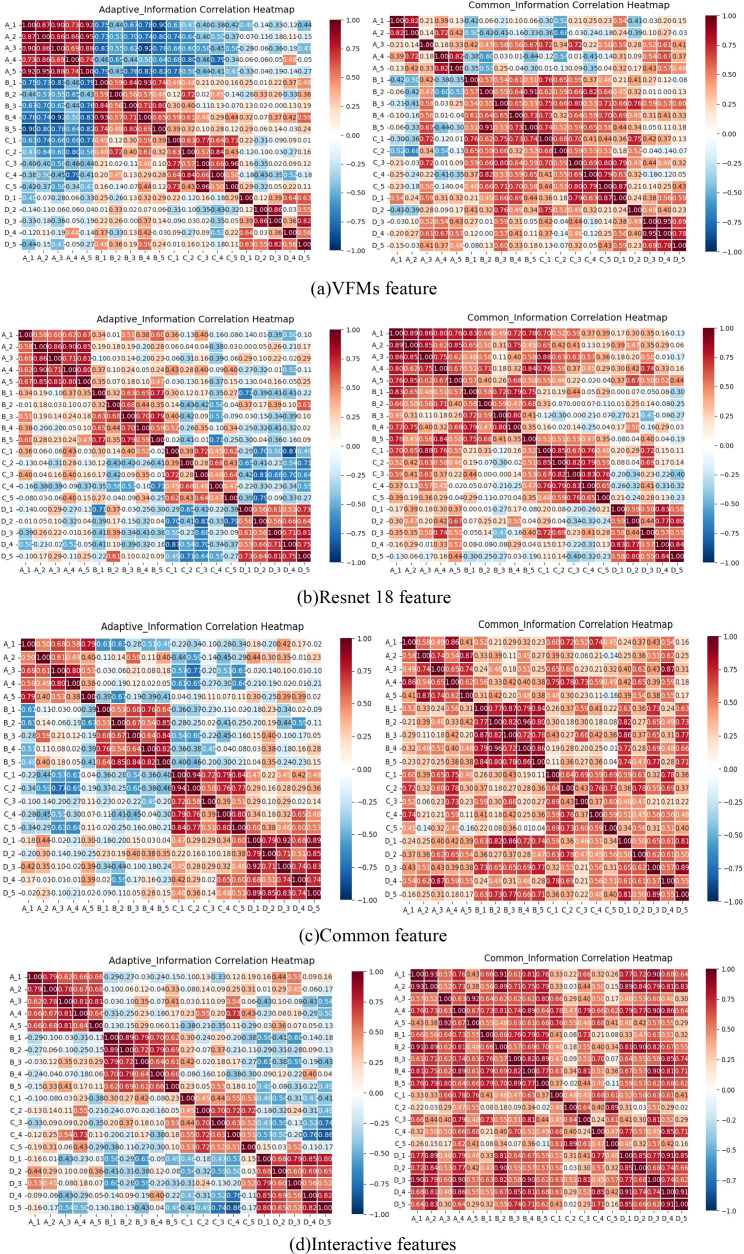
Presents a heat map illustrating cross-center correlations among four types of features across different centers. Group **(a)** demonstrates VFMs feature correlations in large models; Group **(b)** shows ResNet18 feature correlations between centers; Group **(c)** reveals interaction features between large and small models; Group **(d)** highlights shared features between these models.

As shown in **Figure 8**, the personalized features from the small model (a) exhibit low inter-center correlations, sometimes even negative, indicating their strong sensitivity to local data patterns. Higher inter-center correlation would conversely suggest convergence towards a shared representation. Statistical testing further confirms that the ResNet-based features show a slight but significant cross-center difference (t-test p = 0.0389) with a small effect size (d = 0.367), consistent with the weak divergence observed in the heatmap. This suggests that the basic visual features still retain a slight center-specific shift.

In contrast, the personalized features from the large model (b) generally maintain moderate inter-center correlations due to the structural stability provided by the backbone network, while still capturing center-specific variations. Statistical evidence shows that the VFM features exhibit a non-significant divergence trend (t-test p = 0.157, effect size d = 0.50), indicating their cross-center differences are insufficient to establish statistical significance. As the foundation for shared knowledge and cross-domain generalization, the common features (c) are expected to be highly consistent across centers. The heatmap shows their uniform distribution without obvious structural differences, aligning with the statistically non-significant finding for variation (t-test p = 0.215, effect size d = –0.32). This confirms that the common features effectively capture stable, shared information across centers. The interaction features (d) maintain relatively high correlation with the common features while also reflecting cross-center consistency. Statistical analysis reveals that these synergistic (adaptive) features exhibit very strong cross-center divergence (t = 3.1126, p = 0.0037) with a large effect size (d = 1.03). This matches the distinct block-like separation visible in the heatmap, indicating a strong center-specific structure both visually and statistically. Overall, these interaction features balance stability and diversity, enhancing global generalization while retaining local adaptability.

### Feature representation

As shown in **Table 4**, features derived from single models demonstrated limited diagnostic performance. VFMs achieved stable generalization across centers, while ResNet18 exhibited stronger adaptability to local data distributions. In contrast, common features improved cross-center consistency, achieving higher AUCs compared to single-model features (e.g., Center A: 0.7276 vs ~0.8276). Synergy features, which jointly integrated VFM and ResNet18 representations, further boosted performance in certain centers. Importantly, the gated fusion features achieved the best overall performance across all centers, with AUCs reaching up to 0.9255 and ACCs up to 0.8909. These results demonstrate that adaptive fusion effectively balances global semantic consistency with local diagnostic sensitivity, leading to optimal representation learning.

**Table 4:** Feature representation.

feature classifier	Test	Center A	Center B	Center C	Center D
VFMs features	AUC	0.7276	0.7351	0.7950	0.8298
	ACC	0.7302	0.7872	0.7571	0.8182
Resnet18 features	AUC	0.7210	0.8230	0.8150	0.8511
	ACC	0.7090	0.7553	0.7857	0.7636
Common feature	AUC	0.7939	0.7811	0.8050	0.8590
	ACC	0.7619	0.7766	0.8000	0.8545
Synergy feature	AUC	0.7933	0.8338	0.8200	0.8670
	ACC	0.7249	0.8085	0.7857	0.7818
Gated fusion feature	AUC	**0.8276**	**0.8743**	**0.8883**	**0.9255**
	ACC	**0.8148**	**0.8086**	**0.8286**	**0.8909**

The bold values primarily indicate their status as the maximum values in the comparison results.

### Component ablation analysis

The ablation study of the core components in FedCPI (**Table 5**) further demonstrates the contribution of each module. In the ablation setting, when the LMSF component was removed, features extracted from the trained ResNet18 model were directly used for classification without feature decomposition or structured fusion. When the FACM component was excluded, simple average-based federated aggregation was applied instead of subspace-aware communication.

**Table 5:** ablation experiment.

LMSF	FACM	Test	Center A	Center B	Center C	Center D
✓		AUC	0.7731	0.7689	0.8217	0.8218
		ACC	0.7249	0.7234	0.7429	0.8000
	✓	AUC	0.8082	0.8041	0.8367	0.8191
		ACC	0.7302	0.7340	0.7857	0.8727
✓	✓	AUC	**0.8276**	**0.8743**	**0.8883**	**0.9255**
		ACC	**0.8148**	**0.8086**	**0.8286**	**0.8909**

The bold values primarily indicate their status as the maximum values in the comparison results.

The results show that removing LMSF led to a marked performance drop (e.g., AUC at Center B decreased from 0.8743 to 0.7689), confirming that the large–small model feature decomposition and fusion mechanism is essential for resolving semantic inconsistencies and enhancing discriminative representation learning. Similarly, excluding FACM and replacing it with naive averaging resulted in clear degradation (e.g., AUC at Center C declined from 0.8883 to 0.8367), underscoring the importance of adaptive communication in balancing global generalization with local specificity.

When both LMSF and FACM were integrated, FedCPI consistently achieved the best diagnostic performance across all centers, with the highest results observed at Center D (AUC = 0.9255, ACC = 0.8909). These findings highlight the complementary contributions of LMSF and FACM in ensuring robust convergence and cross-center generalization under heterogeneous conditions.

### Independent validation task

Beyond its performance in early-stage NSCLC risk stratification, we evaluated the FedCPI framework in two additional independent validation experiments: (i) predicting postoperative recurrence of gastric cancer, and (ii) detecting myometrial invasion in MRI-confirmed endometrial carcinoma. These experiments demonstrated that the FedCPI framework not only excels in early-stage NSCLC prediction but also demonstrates strong performance in training models for other tasks.

### Postoperative recurrence prediction in gastric cancer

Gastric cancer is one of the most common malignant tumors worldwide. Surgical resection is considered the primary treatment for advanced gastric cancer (AGC). However, due to the high postoperative recurrence rate, survival outcomes remain unsatisfactory. Identifying high-risk patients prone to recurrence after curative gastrectomy allows for early interventions (such as adjuvant chemotherapy and close follow-up), thereby improving prognosis.

We collected clinical data of gastric cancer patients confirmed by postoperative pathology from four hospitals as the third case study, including a total of 641 cases. At each center, data were randomly divided into training and testing sets at a 6:4 ratio. Based on the FedCPI framework, we trained local models at each center and achieved strong testing performance. The AUC values of the testing sets at each center were 0.7589, 0.8089, 0.9018, and 0.8432, respectively.

**Figure 9:**
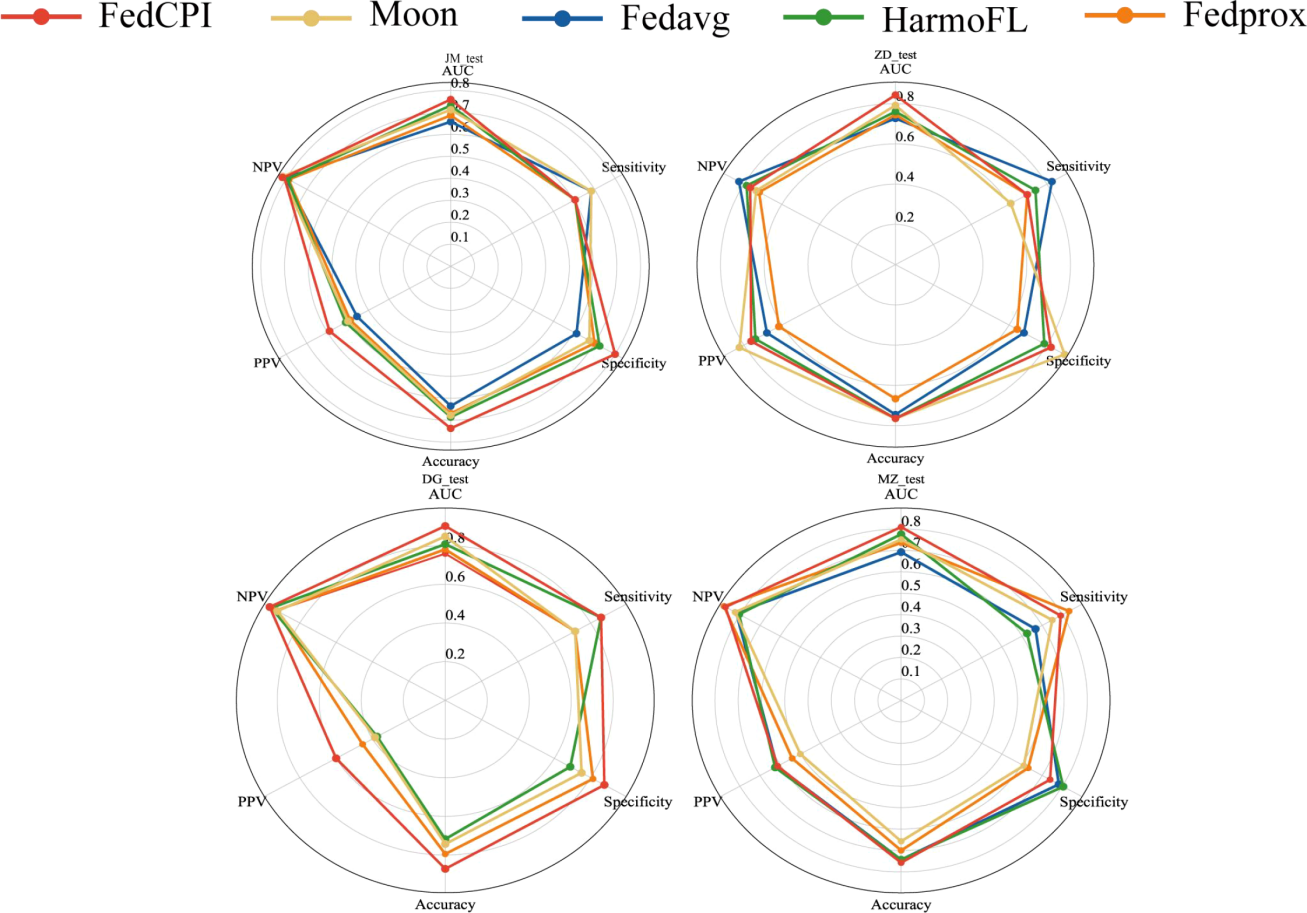
Radar charts for the four centers. Radar chart comparison of five models in four centers.

To assess diagnostic performance, we compared FedCPI with four FL methods. FedCPI achieved average AUC improvement rates of 6.17%, 7.045%, 10.2725%, and 8.7% across the different centers. In addition, FedCPI reached an overall prediction accuracy of 0.7662 (213/278) for identifying recurrence in gastric cancer patients, enabling timely intervention and improved prognosis. Supplementary show the ROC curves and DCA curves of the five methods across four centers. As **Figure 9** illustrated, FedCPI consistently achieved the highest overall performance and net benefit in all four centers. More detailed results are available in **Supplementary S5**.

### Endometrial cancer is distinguished from myometrial invasion on MRI

Endometrial cancer (EC) is one of the most common malignant tumors of the female reproductive system and the sixth leading cause of cancer-related deaths in women. Determining whether myometrial invasion (MI) is present is an important prognostic factor for EC. We collected real-world EC data from five hospitals as the second case study, including 1,186 patients who underwent total hysterectomy for EC. At each center, data were randomly divided into training and testing sets at a 6:4 ratio using random seeds (see **Supplementary Table S6**). Based on the FedCPI framework, we trained local models at each center and achieved promising testing performance. The AUC values of the testing sets at each center were 0.8110, 0.8977, 0.8872, 0.9203, and 0.8700, respectively.

**Figure 10:**
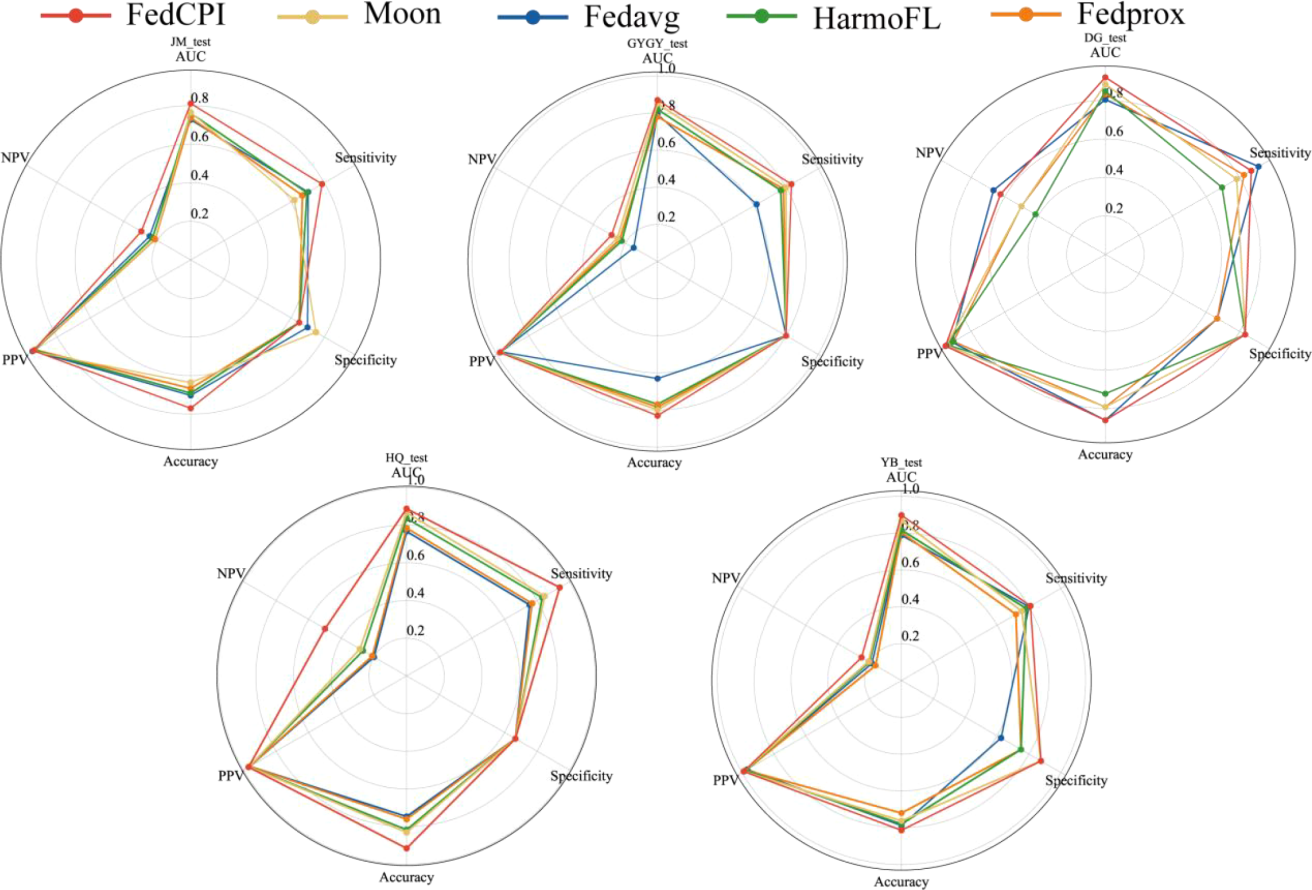
Radar charts for the five centers. Radar chart comparison of five models in four centers.

To evaluate the diagnostic performance of FedCPI, we compared it with four federated learning (FL) methods. FedCPI achieved average AUC improvement rates of 6.3650%, 7.8700%, 7.3725%, 7.7925%, and 5.9175% across different centers. Moreover, FedCPI reached an overall prediction accuracy of 0.8058 (361/448) in distinguishing EC with myometrial invasion, thereby improving the rate of early diagnosis and offering hope for fertility preservation and better prognosis. Figures show the ROC curves and decision curve analysis (DCA) curves of the five methods across four centers. As shown **Figure 10**, FedCPI achieved the highest overall performance and net benefit in all four centers. More detailed results can be found in **Supplementary S6**.

## Discussion

Accurate risk stratification is central to precision oncology, enabling clinicians to tailor treatment intensity and surveillance strategies according to individualized prognostic profiles. However, guideline-based stratification methods remain limited in their ability to delineate subtle yet clinically meaningful risk differences, often resulting in overtreatment of low-risk patients and insufficient intervention for high-risk individuals. These shortcomings underscore the need for advanced, non-invasive, and scalable risk assessment tools capable of capturing complex imaging-derived patterns that reflect underlying tumor biology. To address this challenge, we proposed FedCPI, a federated learning framework that integrates feature-level decomposition with adaptive communication to achieve scale-aware collaboration and subspace-consistent knowledge transfer across heterogeneous medical centers.

### Overall performance and clinical implications

In the core task of early-stage non-small cell lung cancer risk stratification, the FedCPI framework demonstrated highly accurate and robust predictive performance across four independent medical centers. FedCPI significantly outperformed traditional clinical models based on TNM staging, imaging features, and multivariable logistic regression across all centers in terms of model performance metrics, an advantage statistically confirmed by DeLong tests. Furthermore, consistent and significant improvements in Net Reclassification Improvement (NRI) and Integrated Discrimination Improvement (IDI) further validated its exceptional clinical risk reclassification capability. Compared with existing mainstream federated learning approaches (such as FedAve, FedProx, MOON, and HL), FedCPI demonstrated superior or comparable predictive performance in most centers and exhibited a more consistent trend of statistically significant improvements. Although the direct differences in AUC did not reach statistical significance in DeLong tests with some federated learning methods at certain centers (e.g., Centers A and C), FedCPI still showed clear improvement trends or significant gains in key evaluation metrics (particularly NRI and IDI), demonstrating the effectiveness of its methodological design.

Integrating these results, the risk scores generated by the FedCPI framework show promise as reliable biomarkers for prognostic assessment. Their stability, validated across multiple centers, provides a powerful tool for clinical decision-making. The model can accurately identify high-risk patients to optimize adjuvant therapy while avoiding overtreatment of low-risk individuals, thereby strongly advancing the implementation of personalized precision medicine.

### Methodological adaptability, and rationale

Data imbalance and heterogeneity in multi-center settings represent common engineering challenges in medical imaging research. The value of an excellent general framework lies in its ability to address a class of problems (e.g., multi-center data heterogeneity) rather than a single task (e.g., a specific cancer diagnosis). To rigorously evaluate the methodological generalizability of the FedCPI framework in multi-center heterogeneous clinical scenarios, we designed the NSCLC (non-small cell lung cancer), gastric cancer, and endometrial cancer tasks as independent validation tasks. Each disease cohort independently constructed its dataset and trained its specific model. Experimental results demonstrate that the FedCPI framework can be successfully deployed across different validation tasks, exhibiting excellent and robust performance at all participating centers. The models trained using this framework on multiple independent disease cohorts achieved outstanding AUC metrics on the test sets: NSCLC AUC reached 0.9203, endometrial cancer AUC reached 0.9255, and gastric cancer AUC reached 0.9018. This study aims to validate the applicability of the FedCPI framework in diverse data scenarios, demonstrating its ability to learn task-relevant features from multi-center data.

### Mechanistic explanation of performance

The exceptional performance of FedCPI stems from its two core modules: the Large-Small Model Feature Decomposition and Fusion (LMSF) module and the Federated Adaptive Communication Mechanism (FACM). Features from large models (visual foundation models, VFMs) exhibit high intra-group consistency and stability, while features from small models (ResNet18) demonstrate greater variability and flexibility in adapting to local data. There exists complementarity between large and small model features. Visualization analysis via t-SNE of the subspaces decomposed by LMSF reveals that common features form tightly clustered distributions in the central region, indicating a semantic space suitable for unified aggregation; personalized features show distinct center-dependent variations, making them suitable for local retention; and interactive features form structured clusters across centers, achieving a balance between global consistency and local specificity. This decomposition mechanism avoids the information blurring issues often caused by traditional simple pooling or coarse alignment. By dynamically weighting and integrating these subspaces, the resulting representations maintain global consistency, local discriminability, and cross-model complementarity.

The Federated Adaptive Communication Mechanism (FACM) further ensures that the representational advantages of LMSF are preserved during aggregation. FACM employs subspace-specific communication rules—applying global averaging to the common subspace, implementing local retention strategies for the personalized subspace, and adopting performance-driven weighting for the interactive subspace—thereby balancing global generalization with local adaptation. Cross-center correlation analysis reveals that personalized features exhibit low or even negative correlations between different centers, reflecting their high sensitivity to unique local data patterns. In contrast, features generated by VFMs maintain moderate stability across centers. Common features consistently show high correlations across centers, confirming their role as the core foundation of shared knowledge. Interactive features skillfully balance stability and diversity, fully demonstrating their value as a bridge for collaborative knowledge exchange.

### Diagnostic performance and ablation validation

Quantitative results demonstrate that in the analysis and integration of large and small model features, comparing the performance of features from large-scale VFMs, ResNet18, and their shared and interactive features reveals the limited diagnostic capability of single-model features. In contrast, the gated fusion mechanism achieved optimal performance across all centers, with an AUC value as high as 0.9255 and an accuracy of 0.8909, fully validating the effectiveness of adaptive fusion techniques in balancing global semantic stability and local discriminative sensitivity. Ablation studies confirmed the complementary roles of LMSF and FACM: removing LMSF (i.e., bypassing feature decomposition and fusion) led to a significant performance drop (e.g., Center B AUC decreased sharply from 0.8743 → 0.7689). Similarly, individually eliminating FACM and adopting simple averaging also impaired performance (e.g., Center C AUC declined from 0.8883 → 0.8367). Only when LMSF and FACM worked synergistically did FedCPI achieve optimal performance, attaining maximum accuracy improvements of 15–20% across centers. These results indicate that FedCPI not only mitigates data heterogeneity but also transforms it into a valuable source of knowledge, starkly contrasting with traditional federated learning methods such as FedAvg, FedProx, HarmoFL, and Moon. FedCPI achieved improvements of 15.95% and 12.05% in three clinical tasks, with AUC metrics reaching 12.05%, maximum accuracy improvements of 16.4%, 20%, and 15.39%, respectively. ROC curve and decision curve analyses further confirmed that FedCPI delivers stronger net clinical benefits across different medical centers, robustly demonstrating its effectiveness in heterogeneous multicenter environments.

### Limitations and future directions

Despite these encouraging results, several limitations warrant attention. First, larger and more diverse datasets are needed to validate the model’s generalizability across multiple diseases and imaging modalities. Second, although structured feature decomposition enhances interpretability, the uniqueness of subspace partitioning cannot be fully guaranteed, and further research is required to optimize the interpretability framework. Third, while the Federated Adaptive Collaborative Model (FACM) balances global and local adaptation, more sophisticated aggregation strategies need to be developed to address extreme data imbalance scenarios. In response to potential privacy leakage risks in federated learning, this study proposes that targeted protective strategies should be formulated. Future work integrating multi-omics or molecular data holds promise for simultaneously enhancing the model’s biological interpretability and clinical utility.

## Conclusion

In summary, FedCPI represents a robust and versatile federated learning framework that integrates feature decomposition and adaptive communication to deliver accurate risk stratification in heterogeneous multicenter environments. Supported by correlation analyses, t-SNE visualization, cross-center feature studies, and ablation experiments, FedCPI demonstrated superior diagnostic performance not only in early-stage lung cancer but also across gastric and endometrial cancers. Its ability to balance global semantic consistency with local diagnostic sensitivity highlights its promise as a generalizable tool for individualized therapy and prognostic decision-making in oncology.

## Data Availability

This dataset contains protected health information and is not publicly available; use is restricted to the approved research team. Any request for secondary use must obtain prior approval from the Medical Ethics Committee of Jiangmen Central Hospital and execute a Data Use Agreement to ensure compliance with all applicable privacy regulations. Requests to access these datasets should be directed to XC (13^th^ Author) 3897001254@qq.com.

## References

[1] Zeng H Chen W Zheng R Zhang S Ji JS Zou X . Changing cancer survival in China during 2003–15: a pooled analysis of 17 population-based cancer registries. Lancet Global Health. (2018) 6:e555–e567. doi: 10.1016/S2214-109X(18)30127-X, PMID: 29653628

[2] Chinese Thoracic Society . Chinese expert consensus on diagnosis of early lung cancer (2023 Edition). Chin J tuberculosis Respir Dis. (2023) 46:1–18. doi: 10.3760/cma.j.cn112147-20220712-00592, PMID: 36617923

[3] Ardila D Kiraly AP Bharadwaj S Choi B Reicher JJ Peng L . End-to-end lung cancer screening with three-dimensional deep learning on low-dose chest computed tomography. Nat Med. (2019) 25:954–61. doi: 10.1038/s41591-019-0447-x, PMID: 31110349

[4] Hissong E Rao R . Pneumocytoma (sclerosing hemangioma), a potential pitfall. Diagn cytopathology. (2017) 45:744–9. doi: 10.1002/dc.23720, PMID: 28398699

[5] Feng B Shi J Huang L Yang Z Feng S Li J . Robustly federated learning model for identifying high-risk patients with postoperative gastric cancer recurrence. Nat Commun. (2024) 15:742. doi: 10.1038/s41467-024-44946-4, PMID: 38272913 PMC10811238

[6] Lu S Chen Y Chen Y Li P Sun J Zheng C . General lightweight framework for vision foundation model supporting multi-task and multi-center medical image analysis. Nat Commun. (2025) 16:2097. doi: 10.1038/s41467-025-57427-z, PMID: 40025028 PMC11873151

[7] Chen C Chen J Xia L . Artificial intelligence promotes clinical application and research of medical imaging. Radiol Pract. (2024) 01):12–6. doi: 10.13609/j.cnki.1000-0313.2024.01.003

[8] Rieke N Hancox J Li W . The future of digital health with federated learning. NPJ Digit. Med. (2020) 3:119. doi: 10.1038/s41746-020-00323-1, PMID: 33015372 PMC7490367

[9] Zenk M Baid U Pati S . Towards fair decentralized benchmarking of healthcare AI algorithms with the Federated Tumor Segmentation (FeTS) challenge. Nat Commun. (2025) 16:6274. doi: 10.1038/s41467-025-60466-1, PMID: 40628696 PMC12238412

[10] Andreux M du Terrail JO Beguier C Tramel EW . Siloed federated learning for multi-centric histopathology datasets. In: Albarqouni S , editors. Domain Adaptation and Representation Transfer, and Distributed and Collaborative Learning. DART DCL 2020, vol. 12444 Lecture Notes in Computer Science. Springer, Cham (2020). doi: 10.1007/978-3-030-60548-3_13

[11] Wang Z Wang Y Liao X Ming X . FedIFL: A federated cross-domain diagnostic framework for motor-driven systems with inconsistent fault modes. arXiv. (2024). doi: 10.48550/arXiv.2505.07315

[12] Wu C Yin S Qi W Wang . Visual chatGPT: talking, drawing and editing with visual foundation models. arXiv. (2023), abs/2303.04671. doi: 10.48550/arXiv.2303.04671

[13] Zhang H Huang J . Challenging GPU dominance: When CPUs outperform for on-device LLM inference. arXiv:2505.06461. (2024) 16:2097. doi: 10.48550/arXiv.2505.06461

[14] Pan W Xu Z Rajendran S Wang F . An adaptive federated learning framework for clinical risk prediction with electronic health records from multiple hospitals. Patterns. (2024) 5:100898. doi: 10.1016/j.patter.2023.100898, PMID: 38264713 PMC10801228

[15] Hu Z Wang J Mańdziuk J Ren Z Pal NR . Unsupervised feature selection for high-order embedding learning and sparse learning. IEEE Trans Cybernetics. (2025) 55:2355–68. doi: 10.1109/TCYB.2025.3546658, PMID: 40106243

[16] Sun L Zhang W Liu Y . (2024). Research on multimodal data fusion and analysis methods driven by knowledge graph, in: 2024 IEEE 4th International Conference on Data Science and Computer Application (ICDSCA), Dalian, China: IEEE. pp. 898–903. doi: 10.1109/ICDSCA63855.2024.10859319

[17] Zhou H Zhou F Chen H . Cohort-individual cooperative learning for multimodal cancer survival analysis. IEEE Trans Med Imaging. (2025) 44:656–67. doi: 10.1109/TMI.2024.3455931, PMID: 39240739

[18] Zhang Y Xu Y Chen J Xie F Chen H . (2024). Prototypical information bottlenecking and disentangling for multimodal cancer survival prediction. ArXiv. 2024:abs/2401.01646. Available online at: https://api.semanticscholar.org/CorpusID:266741497.

[19] Pervej MF Jin R Dai H . Hierarchical federated learning in wireless networks: pruning tackles bandwidth scarcity and system heterogeneity. IEEE Trans Wireless Commun. (2024) 23:11417–1143. Available online at: https://arxiv.org/abs/2308.01562.

[20] Kang Z Xiao E Li Z Wang L . Deep learning based on resNet-18 for classification of prostate imaging-reporting and data system category 3 lesions. Acad Radiol. (2024) 31:2412–23. doi: 10.1016/j.acra.2023.12.042, PMID: 38302387

[21] Azuma C Ito T Shimobaba T . Adversarial domain adaptation using contrastive learning. Eng Appl Artif Intell. (2023) 123:106394. doi: 10.1016/j.engappai.2023.106394

[22] Bradley AP . The use of the area under the ROC curve in the evaluation of machine learning algorithms. Pattern Recognition. (1997) 30:1145–59. doi: 10.1016/S0031-3203(96)00142-2

[23] Sahu AK Li T Sanjabi M Zaheer M Talwalkar A Smith V . Federated optimization in heterogeneous networks. arXiv. (2018). doi: 10.48550/arXiv.1812.06127

[24] McMahan B Moore E Ramage D Hampson S Arcas BAY . Communication-efficient learning of deep networks from decentralized data. Proceedings of the 20th International Conference on Artificial Intelligence and Statistics, in Proceedings of Machine Learning Research (2017) 54:1273–1282. doi: 10.48550/arXiv.1602.05629

[25] Jiang M Wang Z Dou Q . HarmoFL: harmonizing local and global drifts in federated learning on heterogeneous medical images. Proceedings of the AAAI Conference on Artificial Intelligence (2021) 36:1087–1095. doi: 10.1609/aaai.v36i1.19993

[26] Li Q He B Song D . (2021). Model-contrastive federated learning, in: 2021 IEEE/CVF Conference on Computer Vision and Pattern Recognition (CVPR). Nashville, TN, USA: IEEE. pp. 10708–17. doi: 10.1109/CVPR46437.2021.01057

[27] Vickers AJ Elkin EB . Decision curve analysis: a novel method for evaluating prediction models. Med decision making. (2006) 26:565–74. doi: 10.1177/0272989X06295361, PMID: 17099194 PMC2577036

[28] Dudáš A . Graphical representation of data prediction potential: correlation graphs and correlation chains. Vis Comput. (2024) 40:6969–82. doi: 10.1007/s00371-023-03240-y

[29] Kohavi R . (1995). A study of cross-validation and bootstrap for accuracy estimation and model selection. Proceedings of the 14th international joint conference on Artificial intelligence. Montreal, QC, Canada: Morgan Kaufmann Publishers Inc. 2:1137–1143.

[30] Gu Z Eils R Schlesner M . Complex heatmaps reveal patterns and correlations in multidimensional genomic data. Bioinf (Oxford England). (2016) 32:2847–9. doi: 10.1093/bioinformatics/btw313, PMID: 27207943

