# Supplementary material for: Risk stratification for early-stage NSCLC progression: a federated learning framework with large-small model synergy
Source: Front Oncol. 2025 Dec 16;15:1719433. doi: 10.3389/fonc.2025.1719433 (PMC12750462; doi:10.3389/fonc.2025.1719433)
Supplement: Supplementary file 1 [file DataSheet1.pdf]

S1 Data preparation :

In this study, all original images obtained from CT scans were of a fixed size and exported in the Digital Imaging and Communications in Medicine (DICOM) format. After data collection was completed at each data acquisition center, clinicians screened the data based on inclusion and exclusion criteria in the medical data to obtain clinically significant and high-quality images. Since a single CT scan produces multiple images, with only a few containing lesion-specific layers, it was necessary to filter the patients' CT image data to select those containing lesion-specific images. Additionally, as DICOM format data includes patient privacy information, the original data required anonymization. The preprocessing steps for the medical imaging data in this study were as follows:

(1) Extract patient CT image data from DICOM files to achieve anonymization of patient information.

(2) Clinicians review the patients' CT image data to identify the start, maximum, and end layers of the lesions, as well as their types.

(3) Clinicians delineate the region of interest (ROI) of the lesion from the filtered CT images, defined as a rectangular bounding box containing the entire lesion image.

(4) To meet the input requirements of deep learning models, the images containing lesion ROIs undergo normalization, and the ROI regions are resized to 224×224 pixels.

S2：Experimental equipment and parameters

Statistical analysis was performed using R software (version 4.4.1) and IBM SPSS Statistics 26 software .As for deep learning, the following hardware and software configurations were utilized: Graphics card: NVIDIA RTX A6000、CUDA version 10.2、GPU memory: 48GB、Deep learning framework: PyTorch (GPU version)、Programming language: Python 3.10.13、MATLAB version: 2020b. The parameters for building the deep learning model are detailed in Supplementary Table 1. The pseudocode is provided in Table 2 of the supplementary materials.

Supplementary Table 1 Experimental parameter

learning rate	0.0001	batch size	10
optimizer	SGD	Patch Size	16
momentum	0.9	alpha	0.05
image input shape	3×224×224	weight decay	0.0001
loss function	Cross Entropy	seed	4000
embed_dim	512	depth	20
final_pool_type	mean	channels	3
attention heads	8	Landmarks	256
dim_head	64	Dropout	0.1
Out_dim	256	Transformer（depth）	2
Communication rounds	30	queue size	10
local epoch	1	Pseudo-inverse iterations	6
vfm_dim	786	resnet_dim	512
Common_dim	256	Interaction _dim	256

Supplementary Table 2-Pseudocode

```
Input:
    - K clients with local datasets {D_k}
    - Large model: Vision Foundation Model (VFM)
    - Small model: ResNet18
    - Communication rounds: T
    - Hyperparameters: λ_align, λ_contrast, α (weighting factor)
Initialize:
    - Global ResNet18 backbone Φ_R^0
    - Encoders for subspaces {Φ_C, Φ_P, Φ_I}
For t = 1 to T:    # communication rounds
    --- Local training on each client k ---
    For each client k in parallel:
        1. Feature extraction:
            F_V ← VFM(x)
            F_R ← ResNet18(x)
        2. Feature alignment:
            Compute L_align = || g(F_V) - h(F_R) ||^2
        3. Feature decomposition (LMSF):
            F_C ← Φ_C(F)       # common subspace
            F_P ← Φ_P(F)       # personal subspace (local only)
            F_I ← Φ_I(F)       # interactive subspace
        4. Contrastive learning:
            Apply queue-based contrastive loss L_contrast
        5. Subspace fusion:
            F_fused ← Gating(F_C, F_P, F_I)
            ŷ ← Classifier(F_fused)
        6. Local objective:
```

```
            L_local = L_cls(ŷ, y)
                        + λ_align * L_align
                        + λ_contrast * L_contrast
            Update local parameters
        7. Upload to server:
            {F_C, F_I, ResNet18 weights Φ_R^k, validation score s_k}
            (F_P retained locally)
    --- Server-side aggregation ---
    8. Aggregate common subspace:
        F_C_global = Average(F_C^1, …, F_C^K)
    9. Aggregate interactive subspace:
        w_k = softmax(α * s_k)
        F_I_global = Σ_k w_k * F_I^k
    10. Aggregate ResNet18 backbone:
        Φ_R^t = (1/K) Σ_k Φ_R^{k,t}      # simple average
    11. Distillation and redistribution:
        Construct distillation targets (F_C_global, F_I_global)
        Record contributions (distillation records)
        Send {Φ_R^t, distillation targets, records} to all clients
Output:
    - Global lightweight backbone Φ_R^T
    - Structured encoders {Φ_C, Φ_P, Φ_I}
    - Final FedCPI risk stratification model
```

S3 FesCPI (Federated cross-scale Common–Personal–Interactive learning) Model construction

The large and small model feature fusion framework decouples and fuses two models with different feature representation capabilities. The two types of feature representations refer to the general knowledge representation related to natural images embedded in CT images and the task-specific feature representation. To fully leverage these two types of information, the same CT slice is fed in parallel into the large and small models. This study employs a foundational large vision model (e.g., VFMs) and a lightweight ResNet18 to perform feature extraction and fusion on the same patient's CT slices. Specifically, the local client constructs a ResNet18 model pretrained on the ImageNet dataset and fine-tunes it using corresponding local data to learn feature representations suitable for the task. Simultaneously, a VFMs model built with pretrained parameters and fine-tuned on local data is utilized to learn general feature representations applicable to the task. This study leverages the ResNet18 model's local receptive fields, translation invariance, and hierarchical feature extraction to extract CT image features relevant to the specific medical task. Furthermore, the parameters of a Vision Foundation Model (VFM) pretrained on extensive natural image data are adopted to extract features related to general image information embedded in CT scans, leveraging its capabilities in global context modeling and capturing long-range dependencies.

In cross-domain learning, directly aligning features from different domains can lead to a "semantic gap," often requiring an intermediate shared representation to bridge the domains. Using cross-attention to fuse and align features provides a "bridging semantic space," avoiding feature instability during direct alignment while enhancing the complementarity and clinical interpretability of the large and small models. The module takes $\vec{R}$ and $\vec{V}$ as inputs and computes the attention map H_V of VFM features to ResNet18 features, as well as the attention map H_R of ResNet18 features to VFM features:

$$\begin{aligned} H_V &= soft\max\left(\frac{(\vec{V}U)\times(\vec{R}V)^T}{\sqrt{d}}\right)\in R^{H\times M} \\ H_R &= soft\max\left(\frac{(\vec{R}V)\times(\vec{V}U)^T}{\sqrt{d}}\right)\in R^{M\times H} \end{aligned} \qquad (1)$$

Here, $U\in R^{d\times d}, V\in R^{d\times d}$ is a learnable parameter matrix used to project the original

features, enabling matching in a shared feature space. The attention map H_V reflects the degree of attention VFM features pay to ResNet18 features, while H_R denotes the attention ResNet18 features pay to VFM features. Using these attention distributions, the feature matrix R_V of ResNet18 features relevant to VFM and the feature matrix V_R of VFM features relevant to ResNet18 can be obtained respectively:

$$
\begin{aligned}
R_V &= H_V \times (\vec{R}W_R) \in R^{H \times d} \\
V_R &= H_R \times (\vec{V}W_V) \in R^{M \times d}
\end{aligned}
\tag{2}
$$

Here, $R_V = \{\rho_1, \rho_2, ..., \rho_H\}$, $V_R = \{\varsigma_1, \varsigma_2, ..., \varsigma_M\}$, $W_R \in R^{d \times d}, W_V \in R^{d \times d}$ is a learnable parameter matrix used to adjust the expressive capacity of the feature mapping.

Adopt Sinkhorn divergence, a computationally efficient approximation of optimal transport, as the alignment loss to mitigate distribution discrepancies between small and large model representations as well as across different centers. Specifically, given two feature distributions P and Q, as the alignment loss:

$$L(P,Q) = OT_\varepsilon(P,Q) - \frac{1}{2}(OT_\varepsilon(P,P) + OT_\varepsilon(Q,Q)) \tag{3}$$

where $OT_\varepsilon(*,*)$ denotes the **entropy-regularized optimal transport distance**:

$$OT_\varepsilon(P,Q) = \min_{\pi \in \Pi(P,Q)} \sum_{i,j} \pi_{i,j} c(x_i, y_j) + \varepsilon H(\pi) \tag{4}$$

Here, $\Pi(P,Q)$ is the set of all joint probability couplings with marginals P and Q;

$c(x_i, y_j) = \|x_i - y_i\|^2$ denotes the ground cost between features;

$H(\pi) = \sum_{i,j} \pi_{ij} \log \pi_{ij}$ is the entropy regularization term controlled by $\varepsilon > 0$.

This formulation ensures both **computational efficiency** (thanks to entropy regularization, solvable via Sinkhorn iterations) and **robustness to sample noise**, making it particularly suitable for large-scale federated medical imaging tasks. By minimizing L, the small-model features are encouraged to align with the large-model representation space while still preserving their own local discriminative properties.

Large–Small Model Feature Decomposition and Fusion (LMSF)

The feature embeddings of the two sets of CT images are treated as a CT embedding set.

The CT embedding set for the i-th patient is defined as:

$$\mathrm{F}_{Set(0)}^{i} = \{\mathrm{f}_{(0,1)}^{i}, \mathrm{f}_{(0,2)}^{i}, \ldots, \mathrm{f}_{(0,n)}^{i}\} \in \mathbb{R}^{n \times 512} \tag{5}$$

where n is the number of CT slices.

To further integrate the feature information of the CT scans, a learnable class token $\mathrm{f}_{(0,0)}^{i} \in \mathbb{R}^{1\times512}$ is defined and merged with the CT embedding set, resulting in the overall representation:

$$\mathrm{F}_{Set(0)}^{i} = \{\mathrm{f}_{(0,0)}^{i}, \mathrm{f}_{(0,1)}^{i}, \mathrm{f}_{(0,2)}^{i}, \ldots, \mathrm{f}_{(0,n)}^{i}\} \in \mathbb{R}^{(n+1)\times512} \tag{6}$$

To capture the correlations between CT slices, a self-attention mechanism and a position encoding mechanism are introduced. The integration process is as follows:

$$\begin{aligned} \mathrm{F}_{Set(1)}^{i} &= MSA(LN(\mathrm{F}_{Set(0)}^{i})) + \mathrm{F}_{Set(0)}^{i}, \\ \mathrm{F}_{Set(2)}^{i} &= PPEG(\mathrm{F}_{Set(1)}^{i}), \\ \mathrm{F}_{Set(3)}^{i} &= MSA(LN(\mathrm{F}_{Set(2)}^{i})) + \mathrm{F}_{Set(2)}^{i}, \end{aligned} \tag{7}$$

where MSA denotes the Multi-Head Self-Attention mechanism, LN denotes Layer Normalization, and PPEG denotes the Pyramid Position Encoding Generator module, which is used to encode the positional correlations between CT images. The final output after the above integration is:

$$\mathrm{F}_{Set(3)}^{i} = \{\mathrm{f}_{(3,0)}^{i}, \mathrm{f}_{(3,1)}^{i}, \mathrm{f}_{(3,2)}^{i}, \ldots, \mathrm{f}_{(3,n)}^{i}\} \in \mathbb{R}^{(n+1)\times512} \tag{8}$$

After processing through multiple layers of self-attention and position encoding mechanisms, $\mathrm{f}_{(3,0)}^{i}$ can effectively capture long-range dependencies and local correlations within the CT modality. Therefore, it is taken as the final global feature representation of the CT scan, denoted as $F_{R}^{i} \in \mathbb{R}^{1\times512}$.

This study proposes a feature decomposition and fusion method based on large and small models, with the core idea being to leverage the complementary characteristics of ResNet18 and VFMs (e.g., large models excel at general knowledge, while ResNet models excel at specialized knowledge). Deep mining of CT images is achieved through a multi-level guided feature decomposition module (**LMSF**). This module extracts four distinct feature components from the feature representations F_{V}^{i} and F_{R}^{i} of the large and small models: two are unique features (idiosyncratic features) captured independently by each model, representing characteristics exclusive to that model; common features shared between the models, representing overlapping information which, if not handled properly, could increase redundancy; and emergent features not present in any single model, which are new task-relevant composite information discovered during interaction. Without explicit modeling,

the model might tend to learn more idiosyncratic features and overlook these deeper interactive insights. This comprehensive and explicit division of feature components allows for more effective modeling and utilization of different features, thereby enhancing the model's performance, interpretability, and decision-making capability in cancer prognosis.

The specific implementation is as follows:

To achieve feature decomposition, the module employs four encoders to capture features at different levels from the CT image features of the large and small models. First, MLPs are used to construct the idiosyncratic feature encoders Φ_R and Φ_{V} for the large and small models, respectively, to obtain their unique features, focusing on characteristics that only a specific model is adept at capturing. This can be formulated as:

$$R^i = \Phi_R(F_R^i), P^i = \Phi_{\mathrm{V}}(F_{\mathrm{V}}^i) \tag{9}$$

where $R^i \in \mathbb{R}^{1\times512}$ represents the CT idiosyncratic features of the i-th patient, and $\mathrm{V}^i \in \mathbb{R}^{1\times512}$ represents the pathological idiosyncratic features of the i-th patient. Then, based on a cross-attention mechanism, common feature encoder Enc_C and emergent feature encoder Enc_E are constructed. The weights generated by the cross-attention mechanism serve as a bridge for the interaction between the large and small models, mining and capturing the common features $C^i \in \mathbb{R}^{1\times512}$ and emergent features $E^i \in \mathbb{R}^{1\times512}$ during this interaction. The formulas are expressed as follows:

$$\begin{aligned} A &= f_{\mathrm{V}}{}^T(F_R^i) \cdot f_R(F_{\mathrm{V}}^i) \\ E^i &= Enc_E(F_{\mathrm{V}}^i, F_R^i) = f_A{}^T(A) \cdot F_{\mathrm{V}}^i + f_{A^T}{}^T(A^T) \cdot F_R^i \end{aligned} \tag{10}$$

where A is the co-attention matrix, and $f_{\mathrm{V}}, f_R, f_A, f_{A^T}$ denotes fully connected layers. It is noteworthy that although the common feature encoder Enc_C and the emergent feature encoder Enc_E are constructed similarly, their attention weights differ. The common feature encoder optimizes weights to capture common features between the models, while the emergent feature encoder optimizes weights to capture unique emergent features from the model interaction. Based on these four encoders, the following are extracted from the feature representation F_{V}^i, F_R^i of the large and small models: the idiosyncratic features V^i unique to the large Vision Foundation Model (VFM), the idiosyncratic features R^i unique to the small ResNet model, the common features C^i shared by the models, and the emergent features E^i generated during the interaction between the large and small models.

Due to the high-dimensional redundancy of the input modalities, the features captured by

the encoders might overfit to task-irrelevant information, thereby affecting the model's diagnostic performance. To further enhance the representational capability of each feature type and the model's discriminative power, this study constructs guidance strategies at both the idiosyncratic feature level and the common feature level to fully leverage the heterogeneous information in the data. First, at the idiosyncratic level, the probability distributions of different feature components are analyzed to ensure the model captures discriminative features between the large and small models while suppressing redundant or irrelevant information. Second, at the common level, key differences between diagnostic categories are mined by measuring the probability distributions of patients from different classes, providing guidance for common features to further enhance the model's discriminative ability.

At the idiosyncratic feature level, all feature components are derived from the feature representation $F_{\mathrm{V}}^{i}, F_{R}^{i}$ of the large and small models and maintain unique relationships with it. For instance, idiosyncratic features exist only in the large VFM and do not overlap with features of the small model; common features represent the intersection of the models' features, reflecting shared representations; emergent features are new task-relevant features generated during model interaction, which cannot be obtained from any single model alone. Based on these differences between hierarchical features, this study employs the Wasserstein distance W to calculate the probability distribution differences between various feature types and the feature representation F of the large and small models. Personalized guidance strategies are designed accordingly (Formulas 11-13). The loss function l_k uses W to provide either positive or negative guidance on the feature components, emphasizing the relevance between features and the target modality's features while weakening the influence of irrelevant or interfering information, thereby effectively enhancing the representational capability of each feature type.

$$\begin{aligned} l_k = {} & W\left(F_R^i, R^i\right) + W\left(F_R^i, C^i\right) + W\left(F_V^i, \mathrm{V}^i\right) + W\left(F_V^i, C^i\right) \\ & - W\left(F_R^i, \mathrm{V}^i\right) - W\left(F_V^i, R^i\right) - W\left(F_R^i, \mathrm{E}^i\right) - W\left(F_V^i, \mathrm{E}^i\right) \end{aligned} \tag{11}$$

Notably, F_V^i, F_R^i is detached from the gradient in this process to ensure it does not participate in backpropagation. The hierarchical guidance mechanism is illustrated in

Figure 1.

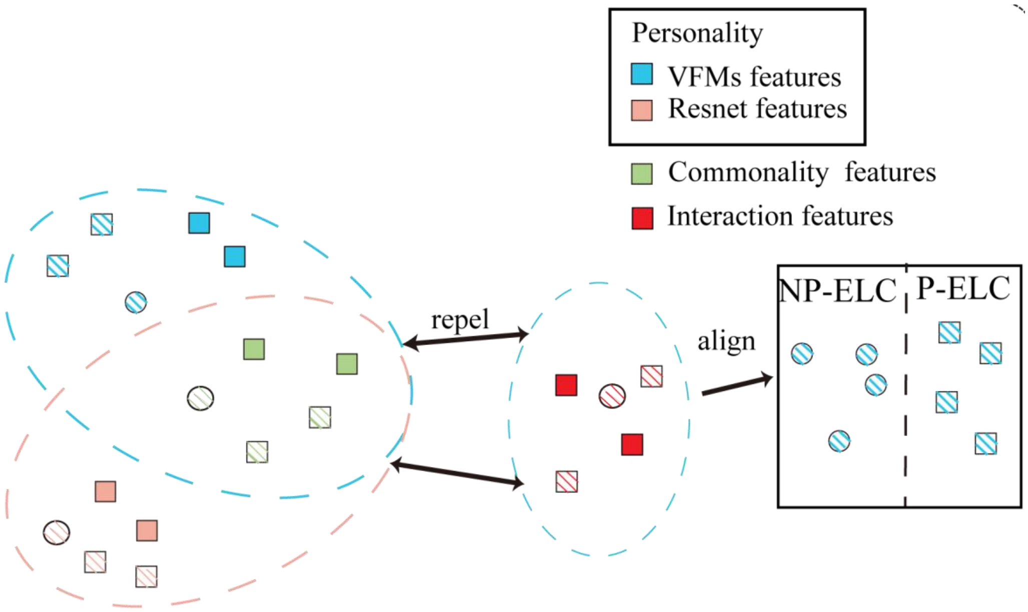

Figure 1 Illustration of the multilevel guidance mechanism

At the shared feature level, the feature representations of patients with disease progression (progressors) are typically similar to those of other progressors and significantly different from those of non-progressors. This assumption aligns with the core idea of contrastive learning. Networks incorporating contrastive learning strategies can effectively extract discriminative feature representations. Therefore, this study proposes a cohort-based contrastive learning method to enhance the model's discriminative ability and generalization performance. Specifically, independent dynamic queues are constructed for each feature type to store the corresponding features of historical patients. Each queue has a length of n and is updated dynamically on a first-in-first-out basis. For the emergent features of a progressor patient, the distance to progressor patients in the emergent feature queue is minimized, while the distance to non-progressor patients is maximized. This makes its features closer to those of same-category patients in the queue and farther from those of different categories. This strategy effectively enhances the representational capability of the emergent features and improves the model's ability to distinguish specific categories. The specific loss design is as follows:

$$l_E = -\log \frac{\sum_{E^{'} \in E_{+}} d\left(E^{i}, E^{'}\right)}{\sum_{E^{'} \in E_{+}} d\left(E^{i}, E^{'}\right) + \sum_{E^{'} \in E_{-}} d\left(E^{i}, E^{'}\right)} \tag{12}$$

where E_+ is the group of category-consistent patients stored in the emergent feature queue, and E_- is the group of category-inconsistent patients. d denotes the Euclidean distance. The guidance process for other features at the patient level is consistent with that for emergent features, so the patient-level loss is $l_v = l_E + l_C + l_R + l_V$.

Combining the guidance learning at both the feature level and the patient level, the feature guidance learning loss can be expressed as:

$$L_{Guidance} = \alpha l_k + \beta l_v \tag{13}$$

where α and β are positive hyperparameters used to adjust the contributions of the feature-level and patient-level loss functions, respectively.

Gated Adaptive Fusion Module Based on Gating Mechanism

In practical applications, CT data from patients at different hospitals exhibit significant variations in information quality, feature distribution, and task relevance. This means the contribution weight of each feature component to the task is not consistent. Fixed weighting or simple concatenation fusion methods often struggle to adapt to these variations, potentially leading to information redundancy or the weakening of critical features. To address this issue, this study designs a Gated Adaptive Fusion Module for the feature fusion stage. The dynamically learns the attention weights for each feature component, adaptively adjusting the importance of different features based on task requirements, thereby achieving effective multi-modal feature fusion. The formulation is as follows:

$$\begin{cases} \omega_k = X_k \bullet W_k^T + b_k \\ \alpha_k = \dfrac{\exp(\omega_k)}{\sum_{j\in\{R,P,C,E\}} \exp(\omega_j)} \\ F_{fused} = \sum\limits_{k\in\{R,P,C,E\}} \alpha_k \bullet X_k \end{cases} \tag{14}$$

The gated adaptive fusion module is a lightweight yet efficient feed-forward neural network, structured as follows: The input to the gating network is the feature vectors $k \in \{R,V,C,E\}$, each with a dimension of 256. These features are fed into a fully connected layer to learn complex interactions among the feature components, outputting an

unnormalized initial score vector ω_k. Here, $W_k \in \mathbb{R}^{256\times1}$ represents the linear transformation weight matrix corresponding to the k-th feature component, and b_k is the bias term. To ensure that all weights sum to 1, a Softmax function is applied to normalize the score vector ω_k, yielding α_k. The final fused feature F_{fused} is the weighted sum of all transformed features, with the weights provided by the gating network.

For the patient pre-operative prediction task, this study uses the cross-entropy loss function L_{Cross} to optimize the model's diagnostic performance. Finally, the overall loss function is:

$$L = L_{Cross} + L_{Guidance} \quad (15)$$

To further enhance the model's predictive performance, this study introduces a Sparse Bayesian Extreme Learning Machine (SBELM) to make predictions based on the fused features F_{fused}. This classifier can effectively capture complex non-linear feature relationships. Furthermore, within the Bayesian framework, it models the hidden layer parameters probabilistically to quantify prediction uncertainty, enhancing the model's robustness to task noise. Given the feature distribution differences between progressor and non-progressor patients, SBELM, with its fast learning and strong generalization capabilities, can further optimize the model's performance in distinguishing between patient categories.

Federated Adaptive Communication Mechanism (FACM)

In the Federated Learning (FL) framework, directly aggregating model parameters often leads to various issues due to the heterogeneous data distributions, inconsistent labels, and divergent task preferences across multiple centers. Particularly in multi-modal, multi-center medical imaging tasks, a single aggregation strategy struggles to simultaneously preserve global collaborative capabilities and local adaptability. Traditional holistic model parameter averaging tends to overlook the differences among center-specific models in terms of structural expression, modal preferences, and task emphasis. To accommodate this heterogeneity, this study proposes a joint optimization strategy that performs federated aggregation and distillation based on knowledge modules. This method effectively balances

global model collaboration and local model personalization, offering an efficient solution for federated learning tasks in cross-center heterogeneous distribution scenarios.

From the perspective of model training, this strategy comprises four core submodules: the local model, the common encoder, the synergy encoder, and the personal knowledge encoder. Each is optimized through differentiated aggregation and module-level distillation mechanisms. The local model and the common encoder employ an average parameter aggregation strategy to extract stable and consistent global feature representations from the shared representations of each center. The synergy encoder adopts a dynamically weighted parameter aggregation mechanism, adjusting the aggregation contribution based on the performance of each center's model on the training set. The personal module does not participate in aggregation and is updated solely through local training and soft distillation to retain local modal features and task preferences. The aggregated global encoder is distributed as a teacher model, providing behavioral guidance to local models via module-level distillation. This enhances their generalization ability while avoiding structural conflicts and catastrophic forgetting. The server builds a global model by integrating the weights of different modules.

Therefore, this study proposes a federated Adaptive Communication Mechanism (FACM), which introduces differentiated aggregation and knowledge distillation between global and local to realize dynamic adaptation across centers. In the average aggregation of common features, the aggregation weight is calculated based on the data volume of each client, and its global model parameters can be defined by the following formula:

$$\theta^{(t+1)} = \sum_{\mathrm{k}=1}^{K} \frac{n_k}{N} \theta_k^{(t)} \tag{16}$$

Here, $\theta_k^{(t)}$ denotes the local model parameters of the k -th client in the t -th iteration, n_k represents the number of samples from the k -th client, and $N = \sum_{\mathrm{k}=1}^{K} n_k$ indicates the total sample count across all centers. This aggregation method ensures that the global model not only retains each center's feature extraction capabilities but also integrates cross-domain knowledge from the Visual Foundation Model (VFM), thereby enhancing the generalization ability of the global model.

For interactive derivative modules, after the t-th round of training, each local server uploads its local model parameters and accuracy $(\omega_i^t, f_{Acci}^{\ t})$ to the central server. The central server then filters the set of qualified local servers D_{Accord} based on a predefined accuracy threshold Q. For the filtered set D_{Accord} the weight for each local server's participation in global model aggregation is calculated as follows:

$$\phi_i^t = \frac{A_i^t}{\sum_{i=1}^{s} A_i^t} \tag{17}$$

where s is the number of models in D_{Accord}; ϕ_i^t and A_i^t are the accuracy and weight of the i-th local server in the t-th round, respectively. Using the parameters ω_i^t of the selected local models and their corresponding calculated weights ϕ_i^t, the global model aggregation is performed via weighted averaging:

$$\omega^{t+1} = \omega^t - \sum_{i=1}^{s}\sum_{i=1}^{s}(\omega_i^t \times \phi_i^t) \tag{18}$$

where ω^t represents the global model parameters for the current t-th round. After the global model aggregation for this round is completed, the central server distributes the global model parameters to all local models that participated in the aggregation. Other local models that did not participate in this round of aggregation retain their own parameters ω_i^t for the round, thereby completing the update for all local models. This process can be expressed as:

$$\begin{cases} \omega_i^{t+1} = \omega^t, D_i \in D_{Accord} \\ \omega_i^{t+1} = \omega_i^t, D_i \notin D_{Accord} \end{cases}, \tag{19}$$

The iteration continues until the predefined number of rounds is reached. The central server then finalizes the global model construction and distributes the final global model parameters to all local servers D_{All}. Each local server D_i initializes its model parameters obtained from the central server and then trains using its local dataset.

By introducing a teacher-student distillation mechanism, the local model parameter updates are guided by the distribution difference between the global model's prediction

probability $p^g(x)$ and the local model's prediction probability $p^l(x)$. The specific loss function is as follows:

$$L_{FACM} = L_{CE}(y, p^l(x)) + \lambda L_{KD}(p^g(x), P^l(x)) \tag{20}$$

where L_{CE} represents the standard cross-entropy loss, measuring the discrepancy between the local model's predictions and the true labels y; L_{KD} represents the distillation loss, often implemented using KL divergence or temperature-scaled cross-entropy:

$$L_{KD}(p^g(x), P^l(x)) = \sum_i p_i^g(x) \log \frac{p_i^g(x)}{p_i^l(x)} \tag{21}$$

where $p^g(x)$ is the prediction distribution of the global model, and $P^l(x)$ is the prediction distribution of the local model. Through joint optimization, the local model can both retain the stability of global knowledge and flexibly adapt according to the characteristics of the local distribution.

At the idiosyncratic feature level, all feature components are derived from the feature representation F_{V}^i, F_R^i of the large and small models and maintain unique relationships with it. For instance, idiosyncratic features exist only in the large VFM and do not overlap with features of the small model; common features represent the intersection of the models' features, reflecting shared representations; emergent features are new task-relevant features generated during model interaction, which cannot be obtained from any single model alone. Based on these differences between hierarchical features, this study employs the Wasserstein distance W to calculate the probability distribution differences between various feature types and the feature representation F of the large and small models. Personalized guidance strategies are designed accordingly (Formulas 11-13). The loss function l_k uses W to provide either positive or negative guidance on the feature components, emphasizing the relevance between features and the target modality's

features while weakening the influence of irrelevant or interfering information, thereby effectively enhancing the representational capability of each feature type.

$$
\begin{aligned}
l_k = & W\left(F_R^i, R^i\right)+W\left(F_R^i, C^i\right)+W\left(F_V^i, \mathrm{V}^i\right)+W\left(F_V^i, C^i\right) \\
& -W\left(F_R^i, \mathrm{V}^i\right)-W\left(F_V^i, R^i\right)-W\left(F_R^i, \mathrm{E}^i\right)-W\left(F_V^i, \mathrm{E}^i\right)
\end{aligned} \tag{11}
$$

Notably, F_V^i, F_R^i is detached from the gradient in this process to ensure it does not participate in backpropagation.

At the shared feature level, the feature representations of patients with disease progression (progressors) are typically similar to those of other progressors and significantly different from those of non-progressors. This assumption aligns with the core idea of contrastive learning. Networks incorporating contrastive learning strategies can effectively extract discriminative feature representations. Therefore, this study proposes a cohort-based contrastive learning method to enhance the model's discriminative ability and generalization performance. Specifically, independent dynamic queues are constructed for each feature type to store the corresponding features of historical patients. Each queue has a length of n and is updated dynamically on a first-in-first-out basis. For the emergent features of a progressor patient, the distance to progressor patients in the emergent feature queue is minimized, while the distance to non-progressor patients is maximized. This makes its features closer to those of same-category patients in the queue and farther from those of different categories. This strategy effectively enhances the representational capability of the emergent features and improves the model's ability to distinguish specific categories. The specific loss design is as follows:

$$
l_E = -\log \frac{\sum_{E^{'} \in E_+} d\left(E^i, E^{'}\right)}{\sum_{E^{'} \in E_+} d\left(E^i, E^{'}\right)+\sum_{E^{'} \in E_-} d\left(E^i, E^{'}\right)} \tag{12}
$$

where E_+ is the group of category-consistent patients stored in the emergent feature queue, and E_- is the group of category-inconsistent patients. d denotes the Euclidean distance. The guidance process for other features at the patient level is consistent with that for emergent features, so the patient-level loss is $l_v = l_E + l_C + l_R + l_V$.

Combining the guidance learning at both the feature level and the patient level, the feature guidance learning loss can be expressed as:

$$L_{Guidance} = \alpha l_k + \beta l_v \tag{13}$$

where α and β are positive hyperparameters used to adjust the contributions of the feature-level and patient-level loss functions, respectively.

Gated Adaptive Fusion Module Based on Gating Mechanism

In practical applications, CT data from patients at different hospitals exhibit significant variations in information quality, feature distribution, and task relevance. This means the contribution weight of each feature component to the task is not consistent. Fixed weighting or simple concatenation fusion methods often struggle to adapt to these variations, potentially leading to information redundancy or the weakening of critical features. To address this issue, this study designs a Gated Adaptive Fusion Module for the feature fusion stage. The dynamically learns the attention weights for each feature component, adaptively adjusting the importance of different features based on task requirements, thereby achieving effective multi-modal feature fusion. The formulation is as follows:

$$\begin{cases} \omega_k = X_k \bullet W_k^T + b_k \\ \alpha_k = \dfrac{\exp(\omega_k)}{\sum_{j\in\{R,P,C,E\}} \exp(\omega_j)} \\ F_{fused} = \sum\limits_{k\in\{R,P,C,E\}} \alpha_k \bullet X_k \end{cases} \tag{14}$$

The gated adaptive fusion module is a lightweight yet efficient feed-forward neural network, structured as follows: The input to the gating network is the feature vectors $k \in \{R,V,C,E\}$, each with a dimension of 256. These features are fed into a fully connected layer to learn complex interactions among the feature components, outputting an unnormalized initial score vector ω_k. Here, $W_k \in \mathbb{R}^{256\times1}$ represents the linear transformation weight matrix corresponding to the k-th feature component, and b_k is the bias term. To ensure that all weights sum to 1, a Softmax function is applied to normalize the score vector ω_k, yielding α_k. The final fused feature F_{fused} is the weighted sum of all transformed features, with the weights provided by the gating network.

For the patient pre-operative prediction task, this study uses the cross-entropy loss function

L_{Cross} to optimize the model's diagnostic performance. Finally, the overall loss function is:

$$L = L_{Cross} + L_{Guidance} \tag{15}$$

To further enhance the model's predictive performance, this study introduces a Sparse Bayesian Extreme Learning Machine (SBELM) to make predictions based on the fused features F_{fused}. This classifier can effectively capture complex non-linear feature relationships. Furthermore, within the Bayesian framework, it models the hidden layer parameters probabilistically to quantify prediction uncertainty, enhancing the model's robustness to task noise. Given the feature distribution differences between progressor and non-progressor patients, SBELM, with its fast learning and strong generalization capabilities, can further optimize the model's performance in distinguishing between patient categories.

Federated Adaptive Communication Mechanism (FACM)

In the Federated Learning (FL) framework, directly aggregating model parameters often leads to various issues due to the heterogeneous data distributions, inconsistent labels, and divergent task preferences across multiple centers. Particularly in multi-modal, multi-center medical imaging tasks, a single aggregation strategy struggles to simultaneously preserve global collaborative capabilities and local adaptability. Traditional holistic model parameter averaging tends to overlook the differences among center-specific models in terms of structural expression, modal preferences, and task emphasis. To accommodate this heterogeneity, this study proposes a joint optimization strategy that performs federated aggregation and distillation based on knowledge modules. This method effectively balances global model collaboration and local model personalization, offering an efficient solution for federated learning tasks in cross-center heterogeneous distribution scenarios.

From the perspective of model training, this strategy comprises four core submodules: the local model, the common encoder, the synergy encoder, and the personal knowledge encoder. Each is optimized through differentiated aggregation and module-level distillation mechanisms. The local model and the common encoder employ an average parameter aggregation strategy to extract stable and consistent global feature representations from the shared representations of each center. The synergy encoder adopts a dynamically weighted parameter aggregation mechanism, adjusting the aggregation contribution based on the performance of each center's

model on the training set. The personal module does not participate in aggregation and is updated solely through local training and soft distillation to retain local modal features and task preferences. The aggregated global encoder is distributed as a teacher model, providing behavioral guidance to local models via module-level distillation. This enhances their generalization ability while avoiding structural conflicts and catastrophic forgetting. The server builds a global model by integrating the weights of different modules.

Therefore, this study proposes a federated Adaptive Communication Mechanism (FACM), which introduces differentiated aggregation and knowledge distillation between global and local to realize dynamic adaptation across centers. In the average aggregation of common features, the aggregation weight is calculated based on the data volume of each client, and its global model parameters can be defined by the following formula:

$$\theta^{(t+1)} = \sum_{k=1}^{K} \frac{n_k}{N} \theta_k^{(t)} \quad (16)$$

Here, $\theta_k^{(t)}$ denotes the local model parameters of the k-th client in the t-th iteration, n_k represents the number of samples from the k-th client, and $N = \sum_{k=1}^{K} n_k$ indicates the total sample count across all centers. This aggregation method ensures that the global model not only retains each center's feature extraction capabilities but also integrates cross-domain knowledge from the Visual Foundation Model (VFM), thereby enhancing the generalization ability of the global model.

For interactive derivative modules, after the t-th round of training, each local server uploads its local model parameters and accuracy $(\omega_i^t, f_{Acci}^{\ t})$ to the central server. The central server then filters the set of qualified local servers D_{Accord} based on a predefined accuracy threshold Q. For the filtered set D_{Accord} the weight for each local server's participation in global model aggregation is calculated as follows:

$$\phi_i^t = \frac{A_i^t}{\sum_{i=1}^{s} A_i^t} \quad (17)$$

where s is the number of models in D_{Accord}; ϕ_i^t and A_i^t are the accuracy and weight of the i-th local server in the t-th round, respectively. Using the parameters ω_i^t of the selected local models and their corresponding calculated weights ϕ_i^t, the global model aggregation is performed via weighted averaging:

$$\omega^{t+1} = \omega^t - \sum_{i=1}^{s}\sum_{i=1}^{s}(\omega_i^t \times \phi_i^t) \quad (18)$$

where ω^t represents the global model parameters for the current t-th round. After the global model aggregation for this round is completed, the central server distributes the global model parameters to all local models that participated in the aggregation. Other local models that did not participate in this round of aggregation retain their own parameters ω_i^t for the round, thereby completing the update for all local models. This process can be expressed as:

$$\begin{cases} \omega_i^{t+1} = \omega^t, D_i \in D_{Accord} \\ \omega_i^{t+1} = \omega_i^t, D_i \notin D_{Accord} \end{cases}, \quad (19)$$

The iteration continues until the predefined number of rounds is reached. The central server then finalizes the global model construction and distributes the final global model parameters to all local servers D_{All}. Each local server D_i initializes its model parameters obtained from the central server and then trains using its local dataset.

By introducing a teacher-student distillation mechanism, the local model parameter updates are guided by the distribution difference between the global model's prediction probability $p^g(x)$ and the local model's prediction probability $p^l(x)$. The specific loss function is as follows:

$$L_{FACM} = L_{CE}(y, p^l(x)) + \lambda L_{KD}(p^g(x), P^l(x)) \quad (20)$$

where L_{CE} represents the standard cross-entropy loss, measuring the discrepancy between the local model's predictions and the true labels y; L_{KD} represents the

distillation loss, often implemented using KL divergence or temperature-scaled cross-entropy:

$$L_{KD}(p^g(x), P^l(x)) = \sum_i p_i^g(x) \log \frac{p_i^g(x)}{p_i^l(x)} \quad (21)$$

where $p^g(x)$ is the prediction distribution of the global model, and $P^l(x)$ is the prediction distribution of the local model. Through joint optimization, the local model can both retain the stability of global knowledge and flexibly adapt according to the characteristics of the local distribution.

S4 Supplement to early lung cancer results

Results of a comparative study of different federal learning of early lung cancer

Table 3 Comparative Performance Across Models

Center	Algorithm	AUC	Accuracy	Sensitivity	Specificity	PPV	NPV
A (JM)	fedavg	0.7642 [0.685-0.843]	0.7090 [0.641-0.769] (134/189)	0.6571 [0.492-0.792] (23/35)	0.7208 [0.645-0.786] (111/154)	0.3485 [0.245-0.469] (23/66)	0.9024 [0.837-0.943] (111/123)
	fedprox	0.7685 [0.691-0.845]	0.6508 [0.580-0.715] (123/189)	0.7143 [0.549-0.837] (25/35)	0.6364 [0.558-0.708] (98/154)	0.3086 [0.219-0.416] (25/81)	0.9074 [0.838-0.949] (98/108)
	HL	0.7787 [0.701-0.856]	0.6878 [0.619-0.750] (130/189)	0.8000 [0.641-0.900] (28/35)	0.6623 [0.585-0.732] (102/154)	0.3500 [0.255-0.459] (28/80)	0.9358 [0.873-0.969] (102/109)
	moon	0.7853 [0.704-0.866]	0.7566 [0.691-0.812] (143/189)	0.6286 [0.463-0.768] (22/35)	0.7857 [0.714-0.843] (121/154)	0.4000 [0.281-0.532] (22/55)	0.9030 [0.841-0.942] (121/134)
	FedCPI	0.8276 [0.755-0.900]	0.8148 [0.753-0.864] (154/189)	0.6857 [0.520-0.814] (24/35)	.8442 [0.779-0.893] (130/154)	0.5000 [0.364-0.636] (24/48)	0.9220 [0.866-0.956] (130/141)
B (SY)	fedavg	0.7642 [0.627-0.902]	0.7447 [0.648-0.822] (70/94)	0.7000 [0.481-0.855] (14/20)	0.7568 [0.648-0.840] (56/74)	0.4375 [0.282-0.607] (14/32)	0.9032 [0.805-0.955] (56/62)
	fedprox	0.7811 [0.636-0.926]	0.7447 [0.648-0.822] (70/94)	0.8000 [0.584-0.919] (16/20)	0.7297 [0.619-0.818] (54/74)	0.4444 [0.295-0.604] (16/36)	0.9310 [0.836-0.973] (54/58)
	HL	0.8095 [0.700-0.919]	0.7128 [0.614-0.794] (67/94)	0.7500 [0.531-0.888] (15/20)	0.7027 [0.591-0.795] (52/74)	0.4054 [0.263-0.565] (15/37)	0.9123 [0.811-0.962] (52/57)
	moon	0.8209 [0.733-0.909]	0.7340 [0.637-0.813] (69/94)	0.8000 [0.584-0.919] (16/20)	0.7162 [0.605-0.806] (53/74)	0.4324 [0.287-0.591] (16/37)	0.9298 [0.833-0.972] (53/57)
	FedCPI	0.8743 [0.798-0.951]	0.8085 [0.717-0.875] (76/94)	0.7000 [0.481-0.855] (14/20)	0.8378 [0.738-0.905] (62/74)	0.5385 [0.355-0.712] (14/26)	0.9118 [0.821-0.959] (62/68)
C (ZD)	fedavg	0.7683 [0.607-0.930]	0.7143 [0.599-0.807] (50/70)	0.7000 [0.397-0.892] (7/10)	0.7167 [0.592-0.815] (43/60)	0.2917 [0.149-0.492] (7/24)	0.9348 [0.825-0.978] (43/46)
	fedprox	0.7767 [0.653-0.900]	0.6857 [0.570-0.782] (48/70)	0.7000 [0.397-0.892] (7/10)	0.6833 [0.558-0.787] (41/60)	0.2692 [0.137-0.461] (7/26)	0.9318 [0.818-0.977] (41/44)
	HL	0.8100	0.8000	0.5000	0.8500	0.3571	0.9107

		[0.682-0.938]	[0.692-0.877] (56/70)	[0.237-0.763] (5/10)	[0.739-0.919] (51/60)	[0.163-0.612] (5/14)	[0.807-0.961] (51/56)
	moon	0.8200 [0.714-0.926]	0.7143 [0.599-0.807] (50/70)	0.9000 [0.596-0.982] (9/10)	0.6833 [0.558-0.787] (41/60)	0.3214 [0.179-0.507] (9/28)	0.9762 [0.877-0.996] (41/42)
	FedCPI	0.8883 [0.812-0.965]	0.8143 [0.708-0.888] (57/70)	0.6000 [0.313-0.832] (6/10)	0.8500 [0.739-0.919] (51/60)	0.4000 [0.198-0.643] (6/15)	0.9273 [0.827-0.971] (51/55)
D (ZS)	fedavg	0.7660 [0.571-0.961]	0.8182 [0.697-0.898] (45/55)	0.6250 [0.306-0.863] (5/8)	0.8511 [0.723-0.926] (40/47)	0.4167 [0.193-0.680] (5/12)	0.9302 [0.814-0.976] (40/43)
	fedprox	0.7660 [0.555-0.977]	0.7455 [0.617-0.842] (41/55)	0.7500 [0.409-0.929] (6/8)	0.7447 [0.605-0.847] (35/47)	0.3333 [0.163-0.563] (6/18)	0.9459 [0.823-0.985] (35/37)
	HL	0.7979 [0.668-0.928]	0.7818 [0.656-0.871] (43/55)	0.8750 [0.529-0.978] (7/8)	0.7660 [0.628-0.864] (36/47)	0.3889 [0.203-0.614] (7/18)	0.9730 [0.862-0.995] (36/37)
	moon	0.8245 [0.716-0.933]	0.7636 [0.637-0.856] (42/55)	0.8750 [0.529-0.978] (7/8)	0.7447 [0.605-0.847] (35/47)	0.3684 [0.191-0.590] (7/19)	0.9722 [0.858-0.995] (35/36)
	FedCPI	0.9255 [0.855-0.996]	0.8909 [0.782-0.949] (49/55)	0.2500 [0.071-0.591] (2/8)	1.0000 [0.924-1.000] (47/47)	1.0000 [0.342-1.000] (2/2)	0.8868 [0.774-0.947] (47/53)

Table 4 Comparison of the significance of each model

FedCIP (rows) / Federated Learning (columns)		Comparison	fedave	fedprox	moon	HL
FedCIP	Center A	DeLong P_FDR	1.184 p=0.2363	1.020 p=0.3078	0.704 p=0.4813	0.905 p=0.3656
		NRI P_FDR	2.210 p=0.0220	0.877 p=0.3460	0.388 p=0.7020	0.948 p=0.3400
		IDI P_FDR	1.509 p=0.1560	1.360 p=0.1560	0.143 p=0.8860	1.699 p=0.1160
	Center B	DeLong P_FDR	1.360 p=0.1739	1.229 p=0.2191	1.004 p=0.3156	1.073, p=0.2831
		NRI P_FDR	3.052 p=0.03	5.310 p<0.001	3.287 P=0.0225	4.505 p<0.001
		IDI P_FDR	3.413 p<0.001	4.462 p<0.001	2.273 P=0.0260	3.708 p<0.001

Center C	DeLong P_FDR	1.242 p=0.2141	1.304, p=0.1924	1.024, p=0.3058	0.954, p=0.3402
	NRI P_FDR	0.875 p=0.3640	1.484, p=0.1520	3.033, p=0.0140	1.191, p=0.2160
	IDI P_FDR	1.722 P=0.0720	1.845, p=0.0780	2.889 p<0.001	1.607, p=0.1240
Center D	DeLong _P_FDR	1.501, p=0.1332	1.377, p=0.1684	1.495, p=0.1349	1.672, p=0.0945
	NRI _P_FDR	6.025, p=0.0000	7.353, p=0.0000	5.113, p=0.0000	6.879 p<0.001
	IDI _P_FDR	1.973, p=0.0360	2.572, p=0.0440	2.425 p<0.001	2.299, p=0.0160

S5 Supplement to gastric cancer results

Results of a comparative study of different federal learning of gastric cancer. As shown in Table 5.

Table 5 Comparative Performance Across Models

**Center**	**Algorithm**	**AUC**	**Accuracy**	**Sensitivity**	**Specificity**	**PPV**	**NPV**
JM	fedavg	0.6579 [0.554-0.762]	0.6271 [0.537-0.709] (74/118)	0.6579 [0.499-0.788] (25/38)	0.6125 [0.503-0.712] (49/80)	0.4464 [0.324-0.576] (25/56)	0.7903 [0.674-0.873] (49/62)
	fedprox	0.6865 [0.580-0.793]	0.6695 [0.580-0.748] (79/118)	0.6053 [0.447-0.744] (23/38)	0.7000 [0.592-0.789] (56/80)	0.4894 [0.353-0.628] (23/47)	0.7887 [0.680-0.868] (56/71)
	HL	0.7319 [0.636-0.827]	0.6780 [0.589-0.756] (80/118)	0.5789 [0.422-0.721] (22/38)	0.7250 [0.619-0.811] (58/80)	0.5000 [0.358-0.642] (22/44)	0.7838 [0.677-0.862] (58/74)
	moon	0.7125 [0.616-0.809]	0.6864 [0.598-0.763] (81/118)	0.6842 [0.525-0.809] (26/38)	0.6875 [0.579-0.778] (55/80)	0.5098 [0.377-0.641] (26/51)	0.8209 [0.713-0.894] (55/67)
	FedCPI	0.7589 [0.663-0.855]	0.7373 [0.651-0.808] (87/118)	0.6053 [0.447-0.744] (23/38)	0.8000 [0.700-0.873] (64/80)	0.5897 [0.434-0.729] (23/39)	0.8101 [0.710-0.881] (64/79)
MZ	fedavg	0.6920 [0.541-0.843]	0.7286 [0.615-0.819] (51/70)	0.5833 [0.388-0.755] (14/24)	0.8043 [0.668-0.893] (37/46)	0.6087 [0.408-0.778] (14/23)	0.7872 [0.651-0.880] (37/47)
	fedprox	0.7355 [0.618-0.853]	0.6857 [0.570-0.782] (48/70)	0.7917 [0.595-0.908] (19/24)	0.6304 [0.486-0.755] (29/46)	0.5278 [0.370-0.680] (19/36)	0.8529 [0.699-0.936] (29/34)
	HL	0.7754 [0.664-0.887]	0.7143 [0.599-0.807] (50/70)	0.5417 [0.351-0.721] (13/24)	0.8043 [0.668-0.893] (37/46)	0.5909 [0.387-0.767] (13/22)	0.7708 [0.635-0.867] (37/48)
	moon	0.7509 [0.621-0.880]	0.6571 [0.540-0.758] (46/70)	0.7500 [0.551-0.880] (18/24)	0.6087 [0.465-0.736] (28/46)	0.5000 [0.345-0.655] (18/36)	0.8235 [0.665-0.917] (28/34)
	FedCPI	0.8089 [0.690-0.928]	0.7714 [0.660-0.854] (54/70)	0.7917 [0.595-0.908] (19/24)	0.7609 [0.621-0.861] (35/46)	0.6333 [0.455-0.781] (19/30)	0.8750 [0.739-0.945] (35/40)
ZD	fedavg	0.7267 [0.579-0.875]	0.7451 [0.611-0.845] (38/51)	0.8261 [0.629-0.930] (19/23)	0.6786 [0.493-0.821] (19/28)	0.6786 [0.493-0.821] (19/28)	0.8261 [0.629-0.930] (19/23)
	fedprox	0.7453 [0.603-0.888]	0.6667 [0.530-0.780] (34/51)	0.6957 [0.491-0.844] (16/23)	0.6429 [0.458-0.793] (18/28)	0.6154 [0.425-0.776] (16/26)	0.7200 [0.524-0.857] (18/25)

	HL	0.7609<br>[0.623-0.899]	0.7451<br>[0.611-0.845]<br>(38/51)	0.6957<br>[0.491-0.844]<br>(16/23)	0.7857<br>[0.605-0.898]<br>(22/28)	0.7273<br>[0.518-0.868]<br>(16/22)	0.7586<br>[0.579-0.878]<br>(22/29)
	moon	0.7919<br>[0.666-0.917]	0.7451<br>[0.611-0.845]<br>(38/51)	0.5217<br>[0.330-0.708]<br>(12/23)	0.9286<br>[0.774-0.980]<br>(26/28)	0.8571<br>[0.601-0.960]<br>(12/14)	0.7027<br>[0.542-0.825]<br>(26/37)
	FedCPI	0.8432<br>[0.733-0.953]	0.7647<br>[0.632-0.860]<br>(39/51)	0.6957<br>[0.491-0.844]<br>(16/23)	0.8214<br>[0.644-0.921]<br>(23/28)	0.7619<br>[0.549-0.894]<br>(16/21)	0.7667<br>[0.591-0.882]<br>(23/30)
DG	fedavg	0.7589<br>[0.566-0.952]	0.7949<br>[0.645-0.892]<br>(31/39)	0.7143<br>[0.359-0.918]<br>(5/7)	0.8125<br>[0.647-0.911]<br>(26/32)	0.4545<br>[0.213-0.720]<br>(5/11)	0.9286<br>[0.774-0.980]<br>(26/28)
	fedprox	0.7813<br>[0.602-0.961]	0.7949<br>[0.645-0.892]<br>(31/39)	0.7143<br>[0.359-0.918]<br>(5/7)	0.8125<br>[0.647-0.911]<br>(26/32)	0.4545<br>[0.213-0.720]<br>(5/11)	0.9286<br>[0.774-0.980]<br>(26/28)
	HL	0.8080<br>[0.576-1.000]	0.7436<br>[0.589-0.854]<br>(29/39)	0.8571<br>[0.487-0.974]<br>(6/7)	0.7188<br>[0.546-0.844]<br>(23/32)	0.4000<br>[0.198-0.643]<br>(6/15)	0.9583<br>[0.798-0.993]<br>(23/24)
	moon	0.8482<br>[0.699-0.998]	0.7436<br>[0.589-0.854]<br>(29/39)	0.7143<br>[0.359-0.918]<br>(5/7)	0.7500<br>[0.579-0.867]<br>(24/32)	0.3846<br>[0.177-0.645]<br>(5/13)	0.9231<br>[0.759-0.979]<br>(24/26)
	FedCPI	0.9018<br>[0.804-0.999]	0.8462<br>[0.703-0.928]<br>(33/39)	0.5714<br>[0.250-0.842]<br>(4/7)	0.9063<br>[0.758-0.968]<br>(29/32)	0.5714<br>[0.250-0.842]<br>(4/7)	0.9063<br>[0.758-0.968]<br>(29/32)

S6 Supplement to endometrial carcinoma results

Results of a comparative study of different federal learning of endometrial carcinoma. As shown in Table 6.

Table 6 Comparative Performance Across Models

Center	Algorithm	AUC	Accuracy	Sensitivity	Specificity	PPV	NPV
JM	fedavg	0.7266 [0.607-0.846]	0.7030 [0.629-0.767] (116/165)	0.7034 [0.625-0.772] (102/145)	0.7000 [0.481-0.855] (14/20)	0.9444 [0.884-0.974] (102/108)	0.2456 [0.152-0.371] (14/57)
	fedprox	0.7369 [0.627-0.847]	0.6667 [0.592-0.734] (110/165)	0.6690 [0.589-0.740] (97/145)	0.6500 [0.433-0.819] (13/20)	0.9327 [0.868-0.967] (97/104)	0.2131 [0.129-0.331] (13/61)
	HL	0.7607 [0.648-0.873]	0.6909 [0.617-0.756] (114/165)	0.6966 [0.617-0.766] (101/145)	0.6500 [0.433-0.819] (13/20)	0.9352 [0.872-0.968] (101/108)	0.2281 [0.138-0.352] (13/57)
	moon	0.7652 [0.651-0.879]	0.6364 [0.561-0.706] (105/165)	0.6207 [0.540-0.696] (90/145)	0.7500 [0.531-0.888] (15/20)	0.9474 [0.883-0.977] (90/95)	0.2143 [0.134-0.324] (15/70)
	FedCPI	0.8110 [0.721-0.901]	0.7697 [0.700-0.827] (127/165)	0.7862 [0.713-0.845] (114/145)	0.6500 [0.433-0.819] (13/20)	0.9421 [0.885-0.972] (114/121)	0.2955 [0.182-0.442] (13/44)
YB	fedavg	0.7886 [0.620-0.958]	0.7881 [0.706-0.852] (93/118)	0.8000 [0.716-0.864] (88/110)	0.6250 [0.306-0.863] (5/8)	0.9670 [0.908-0.989] (88/91)	0.1852 [0.082-0.367] (5/27)
	fedprox	0.8034 [0.716-0.891]	0.7203 [0.633-0.793] (85/118)	0.7182 [0.628-0.794] (79/110)	0.7500 [0.409-0.929] (6/8)	0.9753 [0.914-0.993] (79/81)	0.1622 [0.077-0.311] (6/37)
	HL	0.8170 [0.715-0.919]	0.7542 [0.669-0.823] (89/118)	0.7545 [0.666-0.825] (83/110)	0.7500 [0.409-0.929] (6/8)	0.9765 [0.918-0.994] (83/85)	0.1818 [0.086-0.344] (6/33)
	moon	0.8670 [0.749-0.985]	0.7542 [0.669-0.823] (89/118)	0.7455 [0.657-0.818] (82/110)	0.8750 [0.529-0.978] (7/8)	0.9880 [0.935-0.998] (82/83)	0.2000 [0.100-0.359] (7/35)
	FedCPI	0.8977 [0.800-0.996]	0.8051 [0.724-0.866] (95/118)	0.8000 [0.716-0.864] (88/110)	0.8750 [0.529-0.978] (7/8)	0.9888 [0.939-0.998] (88/89)	0.2414 [0.122-0.421] (7/29)
HQ	fedavg	0.7667 [0.572-0.962]	0.7324 [0.619-0.821] (52/71)	0.7385 [0.620-0.830] (48/65)	0.6667 [0.300-0.903] (4/6)	0.9600 [0.865-0.989] (48/50)	0.1905 [0.077-0.400] (4/21)
	fedprox	0.7846	0.7606	0.7692	0.6667	0.9615	0.2105

		[0.558-1.000]	[0.650-0.845]<br>(54/71)	[0.654-0.855]<br>(50/65)	[0.300-0.903]<br>(4/6)	[0.870-0.989]<br>(50/52)	[0.085-0.433]<br>(4/19)
	HL	0.8359<br>[0.691-0.981]	0.8169<br>[0.712-0.890]<br>(58/71)	0.8308<br>[0.722-0.903]<br>(54/65)	0.6667<br>[0.300-0.903]<br>(4/6)	0.9643<br>[0.879-0.990]<br>(54/56)	0.2667<br>[0.109-0.520]<br>(4/15)
	moon	0.8667<br>[0.733-1.000]	0.8310<br>[0.727-0.901]<br>(59/71)	0.8462<br>[0.739-0.914]<br>(55/65)	0.6667<br>[0.300-0.903]<br>(4/6)	0.9649<br>[0.881-0.990]<br>(55/57)	0.2857<br>[0.117-0.546]<br>(4/14)
	FedCPI	0.8872<br>[0.742-1.000]	0.9296<br>[0.846-0.970]<br>(66/71)	0.9385<br>[0.852-0.976]<br>(61/65)	0.8333<br>[0.436-0.970]<br>(5/6)	0.9839<br>[0.914-0.997]<br>(61/62)	0.5556<br>[0.267-0.811]<br>(5/9)
	fedavg	0.7933<br>[0.606-0.981]	0.6308<br>[0.509-0.738]<br>(41/65)	0.6167<br>[0.490-0.729]<br>(37/60)	0.8000<br>[0.376-0.964]<br>(4/5)	0.9737<br>[0.865-0.995]<br>(37/38)	0.1481<br>[0.059-0.325]<br>(4/27)
	fedprox	0.7800<br>[0.529-1.000]	0.7846<br>[0.670-0.867]<br>(51/65)	0.7833<br>[0.664-0.869]<br>(47/60)	0.8000<br>[0.376-0.964]<br>(4/5)	0.9792<br>[0.891-0.996]<br>(47/48)	0.2353<br>[0.096-0.473]<br>(4/17)
GYSY	HL	0.8200<br>[0.711-0.929]	0.7846<br>[0.670-0.867]<br>(51/65)	0.7667<br>[0.646-0.856]<br>(46/60)	1.0000<br>[0.566-1.000]<br>(5/5)	1.0000<br>[0.923-1.000]<br>(46/46)	0.2632<br>[0.118-0.488]<br>(5/19)
	moon	0.8500<br>[0.748-0.952]	0.8000<br>[0.687-0.879]<br>(52/65)	0.8000<br>[0.682-0.882]<br>(48/60)	0.8000<br>[0.376-0.964]<br>(4/5)	0.9796<br>[0.893-0.996]<br>(48/49)	0.2500<br>[0.102-0.495]<br>(4/16)
	FedCPI	0.8700<br>[0.762-0.978]	0.8154<br>[0.704-0.891]<br>(53/65)	0.8167<br>[0.701-0.894]<br>(49/60)	0.8000<br>[0.376-0.964]<br>(4/5)	0.9800<br>[0.895-0.996]<br>(49/50)	0.2667<br>[0.109-0.520]<br>(4/15)
	fedavg	0.8043<br>[0.552-1.000]	0.8276<br>[0.655-0.924]<br>(24/29)	0.8696<br>[0.679-0.955]<br>(20/23)	0.6667<br>[0.300-0.903]<br>(4/6)	0.9091<br>[0.722-0.975]<br>(20/22)	0.5714<br>[0.250-0.842]<br>(4/7)
	fedprox	0.8333<br>[0.641-1.000]	0.7586<br>[0.579-0.878]<br>(22/29)	0.7826<br>[0.581-0.903]<br>(18/23)	0.6667<br>[0.300-0.903]<br>(4/6)	0.9000<br>[0.699-0.972]<br>(18/20)	0.4444<br>[0.189-0.733]<br>(4/9)
DG	HL	0.8478<br>[0.697-0.998]	0.7241<br>[0.543-0.853]<br>(21/29)	0.6957<br>[0.491-0.844]<br>(16/23)	0.8333<br>[0.436-0.970]<br>(5/6)	0.9412<br>[0.730-0.990]<br>(16/17)	0.4167<br>[0.193-0.680]<br>(5/12)
	moon	0.8841<br>[0.751-1.000]	0.7931<br>[0.616-0.902]<br>(23/29)	0.7826<br>[0.581-0.903]<br>(18/23)	0.8333<br>[0.436-0.970]<br>(5/6)	0.9474<br>[0.754-0.991]<br>(18/19)	0.5000<br>[0.237-0.763]<br>(5/10)
	FedCPI	0.9203<br>[0.808-1.000]	0.8621<br>[0.694-0.945]<br>(25/29)	0.8696<br>[0.679-0.955]<br>(20/23)	0.8333<br>[0.436-0.970]<br>(5/6)	0.9524<br>[0.773-0.992]<br>(20/21)	0.6250<br>[0.306-0.863]<br>(5/8)
